# Exploration of Bis-Cinnamido-Polyamines as Intrinsic Antimicrobial Agents and Antibiotic Enhancers

**DOI:** 10.3390/biom13071087

**Published:** 2023-07-07

**Authors:** Melissa M. Cadelis, Jisoo Kim, Florent Rouvier, Evangelene S. Gill, Kyle Fraser, Marie-Lise Bourguet-Kondracki, Jean Michel Brunel, Brent R. Copp

**Affiliations:** 1 Department of Molecular Medicine and Pathology, School of Medical Sciences, The University of Auckland, Private Bag 92019, Auckland 1142, New Zealand; 2 School of Chemical Sciences, The University of Auckland, Private Bag 92019, Auckland 1142, New Zealand; 3 Membranes et Cibles Therapeutiques (MCT), SSA, INSERM, Aix-Marseille Universite, 27 bd Jean Moulin, 13385 Marseille, France; 4 Laboratoire Molécules de Communication et Adaptation des Micro-Organismes, UMR 7245 CNRS, Muséum National d’Histoire Naturelle, 57 Rue Cuvier (C.P. 54), 75005 Paris, France

**Keywords:** indole, potentiator, antimicrobial, polyamine, antibiotics, antifungal agents, structure-activity relationships

## Abstract

The marine natural product ianthelliformisamine C is a bis-cinnamido substituted spermine derivative that exhibits intrinsic antimicrobial properties and can enhance the action of doxycycline towards the Gram-negative bacterium *Pseudomonas aeruginosa*. As part of a study to explore the structure–activity requirements of these activities, we have synthesized a set of analogues that vary in the presence/absence of methoxyl group and bromine atoms and in the polyamine chain length. Intrinsic antimicrobial activity towards *Staphylococcus aureus*, methicillin-resistant *S. aureus* (MRSA) and the fungus *Cryptococcus neoformans* was observed for only the longest polyamine chain examples of non-brominated analogues while all examples bearing either one or two bromine atoms were active. Weak to no activity was typically observed towards Gram-negative bacteria, with exceptions being the longest polyamine chain examples **13f**, **14f** and **16f** against *Escherichia coli* (MIC 1.56, 7.2 and 5.3 µM, respectively). Many of these longer polyamine-chain analogues also exhibited cytotoxic and/or red blood cell hemolytic properties, diminishing their potential as antimicrobial lead compounds. Two of the non-toxic, non-halogenated analogues, **13b** and **13d**, exhibited a strong ability to enhance the action of doxycycline against *P. aeruginosa*, with >64-fold and >32-fold enhancement, respectively. These results suggest that any future efforts to optimize the antibiotic-enhancing properties of cinnamido-polyamines should explore a wider range of aromatic ring substituents that do not include bromine or methoxyl groups.

## 1. Introduction

**Figure 1:**
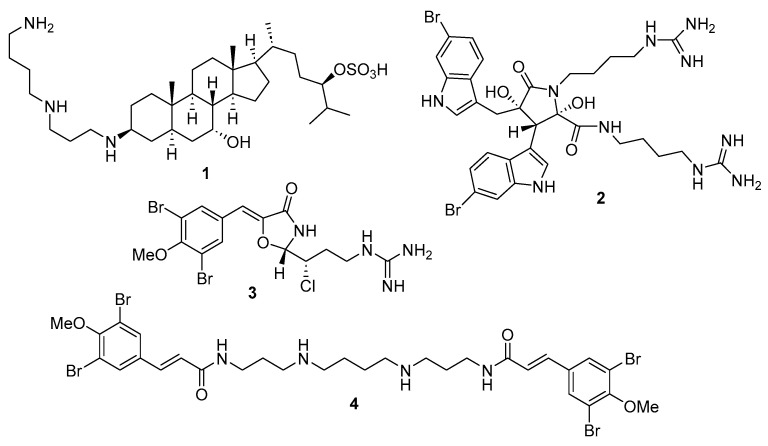
Structures of antimicrobial marine natural products squalamine (**1**), *ent*-eusynstyelamide B (**2**), synoxazolidinone A (**3**) and ianthelliformisamine C (**4**).

With the increasing occurrence of antibiotic-resistant microbial infections, there is an urgent need to discover new classes of antibiotics with new mechanisms of action [1,2]. The marine environment is noted as being a productive source of small molecules, with over 1400 new molecules reported annually [3] and with a sizable percentage of these fitting chemometric descriptors as being drug-like [4]. There are several notable examples of marine natural products that exhibit antimicrobial properties [5] including squalamine (**1**), *ent*-eusynstyelamide B (**2**) and synoxazolidinone A (**3**) (Figure 1). The polyamine-containing aminosterol squalamine (**1**), isolated from the dogfish shark *Squalus acanthias*, exhibits broad-spectrum activity towards both Gram-positive and Gram-negative bacteria [6] and can enhance the action of different classes of antibiotics towards a range of Gram-negative bacteria. Its mechanism of action is attributed to bacterial membrane disruption [7,8] and more recently, to inhibit the glycosyltransferase activity of *Escherichia coli* penicillin-binding protein PBP1b [9]. Knowledge of the structure and attractive biological activities associated with squalamine has led to wide-spread interest in steroidal-polyamine conjugates as a new class of antimicrobial agents [10,11,12]. The discovery that *ent*-eusynstyelamide B (**2**), isolated from the Arctic bryozoan *Tegella* cf. *spitzbergensis* [13], exhibits moderate activity towards Gram-positive bacteria and weaker activity towards Gram-negative bacteria and fungi prompted structure–activity studies that identified examples of easily prepared barbiturate analogues exhibiting potent in vitro and in vivo antibacterial properties [14]. The sub-arctic ascidian-derived natural product synoxazolidinone A (**3**) was originally reported to exhibit modest antimicrobial activity towards *Staphylococcus aureus* [15,16] and that simplification of the structure-identified analogues that could disperse *S. aureus* biofilms and acted synergistically with doxycycline [17].

Investigation of an Australian collection of the marine sponge *Suberea ianthelliformis* led to the characterization of several new polyamine (PA) containing alkaloids including the α,ω-disubstituted spermine (PA-3-4-3) analogue ianthelliformisamine C (**4**) [18]. The natural product demonstrated growth inhibition towards *S. aureus* (MIC 17.5 µM) as well as towards the Gram-negative bacterium *Pseudomonas aeruginosa* with a MIC of 8.75 µM. Two subsequent studies have explored ianthelliformisamine C-related analogues bearing cinnamide head groups with increased bromination and truncated or aryl-containing amine linkers, though no analogues were found to be more active than the natural product [19,20]. A third study [21] reported that ianthelliformisamine C and related analogues were able to enhance the action of legacy antibiotics doxycycline and cefepime toward *P. aeruginosa* PAO1. Intrigued by the antibiotic enhancing properties of **4**, we have prepared a set of new analogues, exploring variation in cinnamate head group substitution as well as variation in polyamine chain length. All analogues were evaluated for antimicrobial activities against a panel of Gram-positive and Gram-negative bacteria and fungi, and for the ability to enhance the antibiotic activity of doxycycline and erythromycin against the Gram-negative bacteria *P. aeruginosa* and *E. coli*, respectively.

## 2. Materials and Methods

### 2.1. Chemistry: General Remarks

Infrared spectra were recorded on a Perkin–Elmer spectrum 100 Fourier Transform infrared spectrometer (PerkinElmer, Boston, MA, USA) equipped with a universal ATR accessory. Mass spectra were acquired on a Bruker micrOTOF Q II spectrometer (Bruker Daltonics, Bremen, Germany). ^1^H and ^13^C NMR spectra were recorded at 298 K on a Bruker AVANCE 400 spectrometer (Bruker, Karlsruhe, Germany) using standard pulse sequences. Proto-deutero solvent signals were used as internal references (DMSO-*d*_6_: δ_H_ 2.50, δ_C_ 39.52; CDCl_3_: δ_H_ 7.26, δ_C_ 77.16; CD_3_OD: δ_H_ 3.31 and δ_C_ 49.0). For ^1^H NMR, the data are quoted as position (δ), relative integral, multiplicity (s = singlet, d = doublet, t = triplet, q = quartet, dd = doublet of doublets, dt = doublet of triplets, tt = triplet of triplets and m = multiplet, br = broad), coupling constant (*J*, Hz) and assignment to the atom. The ^13^C NMR data are quoted as position (δ) and assignment to the atom. Flash column chromatography was carried out using Davisil silica gel (40–60 μm) (Merck, Munich, Germany) or Merck LiChroprep RP-8 (40–63 µm) (Merck, Munich, Germany). Thin layer chromatography was conducted on Merck DC Kieselgel 60 RP-18 F254S plates (Merck, Munich, Germany). All solvents used were of analytical grade or better and/or purified according to standard procedures. Chemical reagents used were purchased from standard chemical suppliers and used as purchased. Ethyl (*E*)-3-(3-bromo-4-methoxyphenyl)acrylate (**9**) [22], 3,5-dibromo-4-methoxybenzaldehyde (**10**) [21], di-*tert*-butyl butane-1,4-diylbis((3-aminopropyl)carbamate) (**12a**), di-*tert*-butyl hexane-1,6-diylbis((3-aminopropyl)carbamate) (**12b**), di-*tert*-butyl heptane-1,7-diylbis((3-aminopropyl)carbamate) (**12c**), di-*tert*-butyl octane-1,8-diylbis((3-aminopropyl)carbamate) (**12d**), di-*tert*-butyl decane-1,10-diylbis((3-aminopropyl)carbamate) (**12e**) and di-*tert*-butyl dodecane-1,12-diylbis((3-aminopropyl)carbamate) (**12f**) [23,24,25,26] were synthesized by literature procedures.

#### 2.1.1. General Procedure A—Amide Bond Formation Variant 1

A solution of carboxylic acid (2.2 equiv.), *N*-(3-dimethylaminopropyl)-*N*′-ethylcarbodiimide hydrochloride (EDC·HCl) (2.6 equiv.), 1-hydroxybenzotriazole (HOBt) (2.6 equiv.) and *N*,*N*-diisopropylethylamine (DIPEA) (6 equiv.) in anhydrous CH_2_Cl_2_ (2 mL) were stirred at 0 °C for 30 min before Boc-protected polyamine **12a**–**f** (1 equiv.) in CH_2_Cl_2_ (1 mL) was added. The reaction mixture was allowed to come to room temperature and stirred under N_2_ atmosphere for 20 h. CH_2_Cl_2_ (30 mL) was added, and the organic solvent layer was washed with sat. aq. NaHCO_3_ (2 × 20 mL) followed by H_2_O (2 × 20 mL), dried under reduced pressure and purified by silica gel column chromatography (2–3% MeOH/CH_2_Cl_2_).

#### 2.1.2. General Procedure B—Amide Bond Formation Variant 2

A solution of carboxylic acid (2.5 equiv.), EDC·HCl (2.8 equiv.) and 4-(dimethylamino)pyridine (DMAP) (5 equiv.) in anhydrous CH_2_Cl_2_ (2 mL) were stirred at 0 °C for 10 min before Boc-protected polyamine **12a**–**f** (1 equiv.) in CH_2_Cl_2_ (1 mL) was added. The reaction mixture was allowed to come to room temperature and stirred under N_2_ atmosphere for 20 h. CH_2_Cl_2_ (30 mL) was added, and the organic solvent layer was washed with sat. aq. NaHCO_3_ (2 × 20 mL) followed by H_2_O (2 × 20 mL), dried under reduced pressure and purified by silica gel column chromatography (2–3% MeOH/CH_2_Cl_2_).

#### 2.1.3. General Procedure C—Boc Deprotection

A solution of *tert*-butyl-carbamate derivative in CH_2_Cl_2_ (2 mL) and trifluoroacetic acid (TFA) (0.2 mL) was stirred at room temperature under N_2_ for 2 h followed by solvent removal under reduced pressure. The crude product was purified using C_8_ reversed-phase flash column chromatography (0–50% MeOH/H_2_O (+0.05% TFA)) to afford the product as a di-TFA salt.

### 2.2. Synthesis of Compounds

#### 2.2.1. (*E*)-3-(3-Bromo-4-methoxyphenyl)acrylic Acid (**7**)

Ethyl (*E*)-3-(3-bromo-4-methoxyphenyl)acrylate (**9**) [22] (451 mg, 1.58 mmol) was dissolved in EtOH (5 mL), and 1N NaOH (10 mL) was added and then heated at reflux under N_2_ for 4 h. EtOH was removed under reduced pressure and the aqueous layer was washed with EtOAc (2 × 10 mL). The aqueous layer was then acidified with 10% HCl (10 mL) and extracted with EtOAc (2 × 10 mL). The combined organic layers were dried with anhydrous MgSO_4_ and the solvent removed under reduced pressure to afford (*E*)-3-(3-bromo-4-methoxyphenyl)acrylic acid (**7**) as a white solid (355 mg, 87%). ^1^H NMR (DMSO-*d*_6_, 400 MHz) δ 12.29 (1H, br s, COOH), 7.96 (1H, d, *J* = 2.1 Hz), 7.70 (1H, dd, *J* = 8.6, 2.1 Hz), 7.51 (1H, d, *J* = 16.0 Hz), 7.14 (1H, d, *J* = 8.6 Hz), 6.46 (1H, d, *J* = 16.0 Hz), 3.89 (3H, s); ^13^C NMR (DMSO-*d*_6_, 100 MHz) 167.6, 156.7, 142.3, 132.5, 129.3, 128.4, 118.1, 112.7, 111.2, 56.5. The ^1^H and ^13^C NMR data agreed with literature values [27].

#### 2.2.2. (*E*)-3-(3,5-Dibromo-4-methoxyphenyl)acrylic Acid (**8**)

To a solution of NaH (60%) (202 mg, 5.0 mmol) in tetrahydrofuran (THF) (5 mL) was added triethyl phosphonoacetate (0.94 mL, 4.7 mmol) at 0 °C and stirred for 45 min. A solution of 3,5-dibromo-4-methoxybenzaldehyde (**10**) [21] (948 mg, 3.2 mmol) in THF (5 mL) was added dropwise and the reaction stirred at rt under N_2_ for 24 h. The crude product mixture was poured onto ice/H_2_O and extracted with EtOAc (2 × 10 mL), the organic layer was washed with brine (20 mL) and was then dried (anhydrous MgSO_4_) and the solvent was removed under reduced pressure. The product was purified by silica gel column chromatography (1% EtOAc/hexane) to afford ethyl (*E*)-3-(3,5-dibromo-4-methoxyphenyl)acrylate (**11**) as a white solid (788 mg, 68%). ^1^H NMR (CDCl_3_, 400 MHz) δ 7.66 (2H, s), 7.50 (1H, d, *J* = 15.8 Hz), 6.36 (1H, d, *J* = 15.8 Hz), 4.26 (2H, q, *J* = 7.3 Hz), 3.91 (3H, s), 1.33 (3H, t, *J* = 7.3 Hz); ^13^C NMR (CDCl_3_, 100 MHz) δ 166.4, 155.6, 141.1, 133.3, 132.1, 120.3, 118.9, 60.9, 14.4. The ^1^H and ^13^C NMR data agreed with those reported in the literature [19]. The product (788 mg, 2.2 mmol) was dissolved in EtOH (5 mL), 1N NaOH (10 mL) added and then heated at reflux under N_2_ for 4 h. EtOH was removed under reduced pressure and the aqueous layer was washed with EtOAc (2 × 10 mL). The aqueous layer was then acidified with 10% HCl (10 mL) and extracted with EtOAc (2 × 10 mL). The combined organic layers were dried with anhydrous MgSO_4_ and the solvent was removed under reduced pressure to afford (*E*)-3-(3,5-dibromo-4-methoxyphenyl)acrylic acid (**8**) as a white solid (632 mg, 86%). ^1^H NMR (DMSO-*d*_6_, 400 MHz) δ 12.48 (1H, br s), 8.05 (2H, s), 7.50 (1H, d, *J* = 16.1 Hz), 6.62 (1H, d, *J* = 15.6 Hz), 3.82 (3H, s); ^13^C NMR (DMSO-*d*_6_, 100 MHz) δ 167.2, 154.4, 140.4, 133.7, 132.3, 121.5, 118.0, 60.5. The ^1^H and ^13^C NMR data agreed with those reported in the literature [19].

#### 2.2.3. *N*^1^,*N*^4^-Bis(3-cinnamamidopropyl)butane-1,4-diaminium 2,2,2-trifluoroacetate (**13a**)

Using general procedure A, reaction of cinnamic acid (**5**) (81 mg, 0.55 mmol), EDC·HCl (124 mg, 0.65 mmol), HOBt (87 mg, 0.64 mmol), DIPEA (0.26 mL, 1.49 mmol) and di-*tert*-butyl butane 1,4-diylbis((3-aminopropyl)carbamate) (**12a**) (100 mg, 0.25 mmol) afforded di-*tert*-butyl butane-1,4-diylbis((3-cinnamamidopropyl) carbamate) as a colorless oil (112 mg, 68%). Using general procedure C, a sub-sample of this product (83 mg, 0.13 mmol) was deprotected to afford the di-TFA salt **13a** as a colorless oil (76 mg, 85%). R*_f_* = 0.46 (MeOH:10% HCl, 7:3); IR (ATR) ν_max_ 3308, 2944, 2833, 1673, 1450, 1116, 1022 cm^−1^; ^1^H NMR (CD_3_OD, 400 MHz) δ 7.59–7.54 (6H, m, H-3, 2H-5), 7.41–7.37 (6H, m, 2H-6, H-7), 6.62 (2H, d, *J* = 15.8 Hz, H-2), 3.44 (4H, t, *J* = 6.4 Hz, H_2_-2′), 3.08–3.04 (8H, m, H_2_-4′, H_2_-6′), 1.96 (4H, tt, *J* = 6.5, 6.5 Hz, H_2_-3′), 1.89–1.81 (4H, m, H_2_-7′); ^13^C NMR (CD_3_OD, 100 MHz) δ 169.7 (C-1), 142.4 (C-3), 136.0 (C-4), 131.1 (C-7), 130.0 (C-6), 128.9 (C-5), 121.2 (C-2), 48.1 (C-6′), 46.3 (C-4′), 37.0 (C-2′), 27.8 (C-3′), 24.3 (C-7′); (+)-HRESIMS [M+H]^+^ *m*/*z* 463.3076 (calcd for C_28_H_39_N_4_O_2_, 463.3068).

#### 2.2.4. *N*^1^,*N*^6^-Bis(3-cinnamamidopropyl)hexane-1,6-diaminium 2,2,2-trifluoroacetate (**13b**)

Using general procedure A, reaction of cinnamic acid (**5**) (37.8 mg, 0.255 mmol), EDC·HCl (57.9 mg, 0.302 mmol), HOBt (40.8 mg, 0.302 mmol), DIPEA (0.12 mL, 0.69 mmol) and di-*tert*-butyl hexane-1,6-diylbis((3-aminopropyl)carbamate) (**12b**) (50 mg, 0.12 mmol) afforded di-*tert*-butyl hexane-1,6-diylbis((3-cinnamamidopropyl)carbamate) as a colorless oil (53 mg, 64%). Using general procedure C, a sub-sample of this product (21 mg, 0.030 mmol) was deprotected to afford the di-TFA salt **13b** as an orange gum (9 mg, 41%). R*_f_* = 0.47 (MeOH:10% HCl, 7:3); IR (ATR) ν_max_ 3747, 3293, 3057, 2866, 1674, 1619, 1553, 1448, 1202, 1133, 721 cm^−1^; ^1^H NMR (CD_3_OD, 400 MHz) δ 7.60–7.54 (6H, m, H-3, 2H-5), 7.42–7.36 (6H, m, 2H-6, H-7), 6.62 (2H, d, *J* = 16.0 Hz, H-2), 3.43 (4H, t, *J* = 6.4 Hz, H_2_-2′), 3.07–3.00 (8H, m, H_2_-4′, H_2_-6′), 1.95 (4H, tt, *J* = 6.5, 6.5 Hz, H_2_-3′), 1.80–1.71 (4H, m, H_2_-7′), 1.52–1.47 (4H, m, H_2_-8′); ^13^C NMR (CD_3_OD, 100 MHz) δ 169.6 (C-1), 142.4 (C-3), 136.1 (C-4), 131.1 (C-7), 130.0 (C-6), 128.9 (C-5), 121.2 (C-2), 48.4 (C-6′), 46.3 (C-4′), 37.0 (C-2′), 27.8 (C-3′), 27.1 (C-7′/C-8′), 27.0 (C-7′/C-8′); (+)-HRESIMS [M+H]^+^ *m*/*z* 491.3374 (calcd for C_30_H_43_N_4_O_2_, 491.3381).

#### 2.2.5. *N*^1^,*N*^7^-Bis(3-cinnamamidopropyl)heptane-1,7-diaminium 2,2,2-trifluoroacetate (**13c**)

Using general procedure A, reaction of cinnamic acid (**5**) (36.7 mg, 0.248 mmol), EDC·HCl (56.1 mg, 0.293 mmol), HOBt (39.6 mg, 0.293 mmol), DIPEA (0.118 mL, 0.677 mmol) and di-*tert*-butyl heptane-1,7-diylbis((3-aminopropyl)carbamate) (**12c**) (50 mg, 0.112 mmol) afforded di-*tert*-butyl heptane-1,7-diylbis((3-cinnamamidopropyl)carbamate) as a colorless oil (36 mg, 45%). Using general procedure C, this product (36 mg, 0.051 mmol) was deprotected to afford the di-TFA salt **13c** as a colorless gum (21 mg, 56%). R*_f_* = 0.47 (MeOH:10% HCl, 7:3); IR (ATR) ν_max_ 3285, 3032, 2939, 1659, 1620, 1556, 1450, 1343, 1286, 1200, 1180, 1131, 980, 834, 800, 767, 721 cm^−1^; ^1^H NMR (CD_3_OD, 400 MHz) δ 7.60–7.54 (6H, m, H-3, 2H-5), 7.42–7.36 (6H, m, 2H-6, H-7), 6.62 (2H, d, *J* = 15.9 Hz, H-2), 3.43 (4H, t, *J* = 6.4 Hz, H_2_-2′), 3.06–2.97 (8H, m, H_2_-4′, H_2_-6′), 1.99–1.90 (4H, m, H_2_-3′), 1.77–1.68 (4H, m, H_2_-7′), 1.49–1.41 (6H, m, H_2_-8′, H_2_-9′); ^13^C NMR (CD_3_OD, 100 MHz) δ 169.6 (C-1), 142.4 (C-3), 136.1 (C-4), 131.0 (C-7), 130.0 (C-6), 128.9 (C-5), 121.2 (C-2), 48.9 (C-6′), 46.3 (C-4′), 37.1 (C-2′), 29.6 (C-9′), 27.7 (C-3′), 27.24 (C-7′/C-8′), 27.15 (C-7′/C-8′); (+)-HRESIMS [M+H]^+^ *m*/*z* 505.3520 (calcd for C_31_H_45_N_4_O_2_, 505.3537).

#### 2.2.6. *N*^1^,*N*^8^-Bis(3-cinnamamidopropyl)octane-1,8-diaminium 2,2,2-trifluoroacetate (**13d**)

Using general procedure A, reaction of cinnamic acid (**5**) (35.5 mg, 0.24 mmol), EDC·HCl (54.3 mg, 0.283 mmol), HOBt (38.3 mg, 0.283 mmol), DIPEA (0.114 mL, 0.654 mmol) and di-*tert*-butyl octane-1,8-diylbis((3-aminopropyl)carbamate) (**12d**) (50 mg, 0.11 mmol) afforded di-*tert*-butyl octane-1,8-diylbis((3-cinnamamidopropyl)carbamate) as a colorless oil (37 mg, 47%). Using general procedure C, a sub-sample of this product (19 mg, 0.026 mmol) was deprotected to afford the di-TFA salt **13d** as a colorless gum (8 mg, 41%). R*_f_* = 0.47 (MeOH:10% HCl, 7:3); IR (ATR) ν_max_ 3284, 2935, 2858, 1674, 1621, 1555, 1451, 1343, 1202, 1133, 980, 845, 800, 768, 721 cm^−1^; ^1^H NMR (CD_3_OD, 400 MHz) δ 7.60–7.54 (6H, m, H-3, 2H-5), 7.43–7.36 (6H, m, 2H-6, H-7), 6.62 (2H, d, *J* = 15.9 Hz, H-2), 3.43 (4H, t, *J* = 6.4 Hz, H_2_-2′), 3.07–2.97 (8H, m, H_2_-4′, H_2_-6′), 1.94 (4H, tt, *J* = 6.5, 6.5 Hz, H_2_-3′), 1.76–1.67 (4H, m, H_2_-7′), 1.47–1.39 (4H, m, H_2_-8′, H_2_-9′); ^13^C NMR (CD_3_OD, 100 MHz) δ 169.6 (C-1), 142.4 (C-3), 136.1 (C-4), 131.1 (C-7), 130.0 (C-6), 128.9 (C-5), 121.2 (C-2), 48.8 (C-6′), 46.3 (C-4′), 37.0 (C-2′), 29.9 (C-9′), 27.8 (C-3′), 27.4 (C-7′/C-8′), 27.3 (C-7′/C-8′); (+)-HRESIMS [M+H]^+^ *m*/*z* 519.3698 (calcd for C_32_H_47_N_4_O_2_, 519.3694).

#### 2.2.7. *N*^1^,*N*^10^-Bis(3-cinnamamidopropyl)decane-1,10-diaminium 2,2,2-trifluoroacetate (**13e**)

Using general procedure A, reaction of cinnamic acid (**5**) (67 mg, 0.45 mmol), EDC·HCl (103 mg, 0.54 mmol), HOBt (72 mg, 0.53 mmol), DIPEA (0.22 mL, 1.26 mmol) and di-*tert*-butyl decane-1,10-diylbis((3-aminopropyl)carbamate) (**12e**) (100 mg, 0.21 mmol) afforded di-*tert*-butyl decane-1,10-diylbis((3-cinnamamidopropyl)carbamate) as a colorless oil (60 mg, 38%). Using general procedure C, a sub-sample of this product (42 mg, 0.056 mmol) was deprotected to afford the di-TFA salt **13e** as a colorless oil (40 mg, 92%). R*_f_* = 0.25 (MeOH:10% HCl, 7:3); IR (ATR) ν_max_ 3311, 2944, 2832, 1683, 1448, 1115, 1022 cm^−1^; ^1^H NMR (CD_3_OD, 400 MHz) δ 7.60–7.54 (6H, m, H-3, 2H-5), 7.43–7.36 (6H, m, 2H-6, H-7), 6.62 (2H, d, *J* = 15.9 Hz, H-2), 3.43 (4H, t, *J* = 6.5 Hz, H_2_-2′), 3.06–2.96 (8H, m, H_2_-4′, H_2_-6′), 1.94 (4H, tt, *J* = 6.5, 6.5 Hz, H_2_-3′), 1.75–1.66 (4H, m, H_2_-7′), 1.46–1.34 (12H, m, H_2_-8′, H_2_-9′, H_2_-10′); ^13^C NMR (CD_3_OD, 100 MHz) δ 169.6 (C-1), 142.4 (C-3), 136.1 (C-4), 131.1 (C-7), 130.0 (C-6), 128.9 (C-5), 121.2 (C-2), 48.8 (C-6′), 46.3 (C-4′), 37.0 (C-2′), 30.4 (C-9′/C-10′), 30.2 (C-9′/C-10′), 27.8 (C-3′), 27.5 (C-7′/C-8′), 27.4 (C-7′/C-8′); (+)-HRESIMS [M+H]^+^ *m*/*z* 547.4022 (calcd for C_34_H_51_N_4_O_2_, 547.4007).

#### 2.2.8. *N*^1^,*N*^12^-Bis(3-cinnamamidopropyl)dodecane-1,12-diaminium 2,2,2-trifluoroacetate (**13f**)

Using general procedure A, reaction of cinnamic acid (**5**) (31.6 mg, 0.213 mmol), EDC·HCl (48.4 mg, 0.253 mmol), HOBt (34.1 mg, 0.252 mmol), DIPEA (0.101 mL, 0.582 mmol) and di-*tert*-butyl dodecane-1,12-diylbis((3-aminopropyl)carbamate) (**12f**) (5.0 mg, 0.0097 mmol) afforded di-*tert*-butyl dodecane-1,12-diylbis((3-cinnamamidopropyl)carbamate as a colorless oil (7.0 mg, 93%). Using general procedure C, this product (7.0 mg, 0.0090 mmol) was deprotected to afford the di-TFA salt **13f** as a colorless gum (4.0 mg, 55%). R*_f_* = 0.16 (MeOH:10% HCl, 7:3); IR (ATR) ν_max_ 3276, 3076, 2926, 2859, 1675, 1621, 1546, 1460, 1202, 1133 cm^−1^; ^1^H NMR (CD_3_OD, 400 MHz) δ 7.61–7.54 (6H, m, H-3, 2H-5), 7.43–7.37 (6H, m, 2H-6, H-7), 6.62 (2H, d, *J* = 15.9 Hz, H-2), 3.43 (4H, t, *J* = 6.5 Hz, H_2_-2′), 3.07–2.96 (8H, m, H_2_-4′, H_2_-5′), 1.94 (4H, tt, *J* = 6.5, 6.5 Hz, H_2_-3′), 1.76–1.65 (4H, m, H_2_-7′), 1.48–1.27 (16H, m, H_2_-8′, H_2_-9′, H_2_-10′, H_2_-11′); ^13^C NMR (CD_3_OD, 100 MHz) δ 169.7 (C-1), 142.5 (C-3), 136.1 (C-4), 131.1 (C-7), 130.0 (C-6), 128.9 (C-5), 121.1 (C-2), 48.8 (C-6′), 46.3 (C-4′), 37.0 (C-2′), 30.7 (C-9′/C-10′/C-11′), 30.5 (C-9′/C-10′/C-11′), 30.2 (C-9′/C-10′/C-11′), 27.8 (C-3′), 27.5 (C-7′/C-8′), 27.3 (C-7′/C-8′); (+)-HRESIMS [M+H]^+^ *m*/*z* 575.4299 (calcd for C_36_H_55_N_4_O_2_, 575.4320).

#### 2.2.9. *N*^1^,*N*^4^-Bis(3-((*E*)-3-(4-methoxyphenyl)acrylamido)propyl)butane-1,4-diaminium 2,2,2-trifluoroacetate (**14a**)

Using general procedure B, reaction of 4-methoxycinnamic acid (**6**) (55 mg, 0.309 mmol), EDC·HCl (67 mg, 0.350 mmol), DMAP (76 mg, 0.622 mmol) and di-*tert*-butyl butane-1,4-diylbis((3-aminopropyl)carbamate) (**12a**) (50 mg, 0.124 mmol) afforded di-*tert*-butyl butane-1,4-diylbis ((3-((*E*)-3-(4-methoxyphenyl)acrylamido)propyl)carbamate) as a white oil (53 mg, 59%). Using general procedure C, a sub-sample of this product (27 mg, 0.037 mmol) was deprotected to afford the di-TFA salt **14a** as a yellow oil (13 mg, 46%). R*_f_* = 0.34 (MeOH:10% HCl, 7:3); IR (ATR) ν_max_ 3247, 3031, 2839, 1673, 1604, 1514, 1200, 1138, 1027, 830, 799, 721 cm^−1^; ^1^H NMR (DMSO-*d*_6_, 400 MHz) δ 8.59 (4H, br s, NH_2_-5′), 8.26 (2H, t, *J* = 5.9 Hz, NH-1′), 7.51 (4H, d, *J* = 8.9 Hz, 2H-5), 7.38 (2H, d, *J =* 15.7 Hz, H-3), 6.97 (4H, d, *J* = 8.9 Hz, 2H-6), 6.48 (2H, d, *J* = 15.7 Hz, H-2), 3.78 (6H, s, OMe), 3.25 (4H, dt, *J* = 6.4, 6.4 Hz, H_2_-2′), 2.96–2.90 (8H, m, H_2_-4′, H_2_-6′), 1.79 (4H, tt, *J* = 7.5, 7.5 Hz, H_2_-3′), 1.66–1.61 (4H, m, H_2_-7′); ^13^C NMR (DMSO-*d*_6_, 100 MHz) δ 165.8 (C-1), 160.4 (C-7), 138.6 (C-3), 129.1 (C-5), 127.3 (C-4), 119.3 (C-2), 114.4 (C-6), 55.3 (OMe), 46.1 (C-6′), 44.7 (C-4′), 35.8 (C-2′), 26.2 (C-3′), 22.7 (C-7′); (+)-HRESIMS [M+H]^+^ *m*/*z* 523.3287 (calcd for C_30_H_43_N_4_O_4_, 523.3279).

#### 2.2.10. *N*^1^,*N*^6^-Bis(3-((*E*)-3-(4-methoxyphenyl)acrylamido)propyl)hexane-1,6-diaminium 2,2,2-trifluoroacetate (**14b**)

Following general procedure B, reaction of 4-methoxycinnamic acid (**6**) (52 mg, 0.292 mmol), EDC·HCl (62 mg, 0.323 mmol), DMAP (71 mg, 0.581 mmol) and di-*tert*-butyl hexane-1,6-diylbis((3-aminopropyl)carbamate) (**12b**) (50 mg, 0.116 mmol) afforded di-*tert*-butyl hexane-1,6-diylbis((3-((*E*)-3-(4-methoxyphenyl)acrylamido)propyl)carbamate) as a white oil (29 mg, 33%). Using general procedure C, a sub-sample of this product was deprotected to afford the di-TFA salt **14b** as a yellow oil (10 mg, 64%). R*_f_* = 0.34 (MeOH:10% HCl, 7:3); IR (ATR) ν_max_ 3285, 2840, 1674, 1603, 1514, 1202, 1175, 1133, 832, 722 cm^−1^; ^1^H NMR (DMSO-*d*_6_, 400 MHz) δ 8.45 (4H, br s, NH_2_-5′), 8.24 (2H, t, *J* = 5.5 Hz, NH-1′), 7.51 (4H, d, *J* = 8.9 Hz, 2H-5), 7.38 (2H, d, *J =* 16.1 Hz, H-3), 6.97 (4H, d, *J* = 9.3 Hz, 2H-6), 6.47 (2H, d, *J* = 15.7 Hz, H-2), 3.79 (6H, s, OMe), 3.25 (4H, dt, *J* = 6.4, 6.4 Hz, H_2_-2′), 2.94–2.86 (8H, m, H_2_-4′, H_2_-6′), 1.83–1.73 (4H, m, H_2_-3′), 1.60–1.54 (4H, m, H_2_-7′), 1.34–1.30 (4H, m, H_2_-8′); ^13^C NMR (DMSO-*d*_6_, 100 MHz) δ 165.8 (C-1), 160.4 (C-7), 138.6 (C-3), 129.1 (C-5), 127.3 (C-4), 119.3 (C-2), 114.4 (C-6), 55.3 (OMe), 46.6 (C-6′), 44.7 (C-4′), 35.8 (C-2′), 26.2 (C-3′), 25.5 (C-7′/C-8′), 25.4 (C-7′/C-8′); (+)-HRESIMS [M+H]^+^ *m*/*z* 551.3593 (calcd for C_32_H_47_N_4_O_4_, 551.3592).

#### 2.2.11. *N*^1^,*N*^7^-Bis(3-((*E*)-3-(4-methoxyphenyl)acrylamido)propyl)heptane-1,7-diaminium 2,2,2-trifluoroacetate (**14c**)

Using general procedure B, reaction of 4-methoxycinnamic acid (**6**) (53 mg, 0.300 mmol), EDC·HCl (65 mg, 0.340 mmol), DMAP (73 mg, 0.600 mmol) and di-*tert*-butyl heptane-1,7-diylbis((3-aminopropyl)carbamate) (**12c**) (50 mg, 0.112 mmol) afforded di-*tert*-butyl heptane-1,7-diylbis((3-((*E*)-3-(4-methoxyphenyl)acrylamido)propyl)carbamate) as a white oil (45 mg, 52%). Using general procedure C, a sub-sample of this product (23 mg, 0.030 mmol) was deprotected to afford the di-TFA salt **14c** as a yellow oil (19 mg, 80%). R*_f_* = 0.34 (MeOH:10% HCl, 7:3); IR (ATR) ν_max_ 3280, 2938, 1657, 1602, 1513, 1200, 1174, 1131, 1028, 830, 721 cm^−1^; ^1^H NMR (DMSO-*d*_6_, 400 MHz) δ 8.61 (4H, br s, NH_2_-5′), 8.29 (2H, t, *J* = 5.7 Hz, NH-1′), 7.51 (4H, d, *J* = 8.8 Hz, 2H-5), 7.38 (2H, d, *J =* 15.8 Hz, H-3), 6.97 (4H, d, *J* = 8.8 Hz, 2H-6), 6.49 (2H, d, *J* = 15.8 Hz, H-2), 3.78 (6H, s, OMe), 3.25 (4H, dt, *J* = 6.4, 6.4 Hz, H_2_-2′), 2.97–2.83 (8H, m, H_2_-4′, H_2_-6′), 1.78 (4H, tt, *J* = 7.5, 7.5 Hz, H_2_-3′), 1.62–1.53 (4H, m, H_2_-7′), 1.33–1.26 (6H, m, H_2_-8′, H_2_-9′); ^13^C NMR (DMSO-*d*_6_, 100 MHz) δ 165.8 (C-1), 160.4 (C-7), 138.6 (C-3), 129.1 (C-5), 127.4 (C-4), 119.4 (C-2), 114.4 (C-6), 55.3 (OMe), 46.7 (C-6′), 44.7 (C-4′), 35.8 (C-2′), 28.0 (C-9′), 26.1 (C-3′), 25.7 (C-7′/C-8′), 25.4 (C-7′/C-8′); (+)-HRESIMS [M+H]^+^ *m*/*z* 565.3748 (calcd for C_33_H_49_N_4_O_4_, 565.3748).

#### 2.2.12. *N*^1^,*N*^8^-Bis(3-((*E*)-3-(4-methoxyphenyl)acrylamido)propyl)octane-1,8-diaminium 2,2,2-trifluoroacetate (**14d**)

Using general procedure B, reaction of 4-methoxycinnamic acid (**6**) (52 mg, 0.292 mmol), EDC·HCl (62 mg, 0.323 mmol), DMAP (71 mg, 0.581 mmol) and di-*tert*-butyl octane-1,8-diylbis((3-aminopropyl)carbamate) (**12d**) (50 mg, 0.109 mmol) afforded di-*tert*-butyl octane-1,8-diylbis((3-((*E*)-3-(4-methoxyphenyl)acrylamido)propyl) carbamate) as a white oil (49 mg, 58%). Using general procedure C, a sub-sample of this product (25 mg, 0.032 mmol) was deprotected to afford the di-TFA salt **14d** as a yellow oil (17 mg, 66%). R*_f_* = 0.26 (MeOH:10% HCl, 7:3); IR (ATR) ν_max_ 3282, 2937, 2840, 1658, 1602, 1513, 1423, 1174, 1130, 1028, 830, 721 cm^−1^; ^1^H NMR (DMSO-*d*_6_, 400 MHz) δ 8.51 (4H, br s, NH_2_-5′), 8.25 (2H, t, *J* = 5.8 Hz, NH-1′), 7.51 (4H, d, *J* = 8.5 Hz, 2H-5), 7.38 (2H, d, *J =* 15.6 Hz, H-3), 6.97 (4H, d, *J* = 8.5 Hz, 2H-6), 6.48 (2H, d, *J* = 15.6 Hz, H-2), 3.78 (6H, s, OMe), 3.25 (4H, tt, *J* = 6.4, 6.4 Hz, H_2_-2′), 2.96–2.83 (8H, m, H_2_-4′, H_2_-6′), 1.78 (4H, tt, *J* = 7.5, 7.5 Hz, H_2_-3′), 1.60–1.52 (4H, m, H_2_-7′), 1.32–1.24 (8H, m, H_2_-8′, H_2_-9′); ^13^C NMR (DMSO-*d*_6_, 100 MHz) δ 165.8 (C-1), 160.4 (C-7), 138.6 (C-3), 129.1 (C-5), 127.3 (C-4), 119.4 (C-2), 114.4 (C-6), 55.3 (OMe), 46.8 (C-6′), 44.7 (C-4′), 35.8 (C-2′), 28.3 (C-9′), 26.2 (C-3′), 25.8 (C-7′/C-8′), 25.5 (C-7′/C-8′); (+)-HRESIMS [M+H]^+^ *m*/*z* 579.3895 (calcd for C_34_H_51_N_4_O_4_, 579.3905).

#### 2.2.13. *N*^1^,*N*^10^-Bis(3-((*E*)-3-(4-methoxyphenyl)acrylamido)propyl)decane-1,10-diaminium 2,2,2-trifluoroacetate (**14e**)

Using general procedure B, reaction of 4-methoxycinnamic acid (**6**) (46 mg, 0.258 mmol), EDC·HCl (55 mg, 0.287 mmol), DMAP (63 mg, 0.516 mmol) and di-*tert*-butyl decane-1,10-diylbis((3-aminopropyl)carbamate) (**12e**) (50 mg, 0.103 mmol) afforded di-*tert*-butyl decane-1,10-diylbis((3-((*E*)-3-(4-methoxyphenyl)acrylamido)propyl) carbamate) as a white oil (30 mg, 36%). Using general procedure C, this product (30 mg, 0.037 mmol) was deprotected to afford the di-TFA salt **14e** as a yellow oil (18 mg, 58%). R*_f_* = 0.19 (MeOH:10% HCl, 7:3); IR (ATR) ν_max_ 3386, 2932, 2855, 1672, 1602, 1513, 1424, 1201, 1133, 1030, 832, 722 cm^−1^; ^1^H NMR (DMSO-*d*_6_, 400 MHz) δ 8.46 (4H, br s, NH_2_-5′), 8.24 (2H, t, *J* = 5.8 Hz, NH-1′), 7.51 (4H, d, *J* = 8.8 Hz, 2H-5), 7.39 (2H, d, *J =* 15.8 Hz, H-3), 6.97 (4H, d, *J* = 8.8 Hz, 2H-6), 6.48 (2H, d, *J* = 15.8 Hz, H-2), 3.78 (6H, s, OMe), 3.25 (4H, dt, *J* = 6.4, 6.4 Hz, H_2_-2′), 2.96–2.84 (8H, m, H_2_-4′, H_2_-6′), 1.78 (4H, tt, *J* = 7.5, 7.5 Hz, H_2_-3′), 1.60–1.52 (4H, m, H_2_-7′), 1.32–1.23 (12H, m, H_2_-8′, H_2_-9′, H_2_-10′); ^13^C NMR (DMSO-*d*_6_, 100 MHz) δ 165.8 (C-1), 160.4 (C-7), 138.6 (C-3), 129.1 (C-5), 127.3 (C-4), 119.3 (C-2), 114.4 (C-6), 55.2 (OMe), 46.8 (C-6′), 44.7 (C-4′), 35.8 (C-2′), 28.7 (C-9′), 28.5 (C-10′), 26.2 (C-3′), 25.9 (C-7′/C-8′), 25.5 (C-7′/C-8′); (+)-HRESIMS [M+H]^+^ *m*/*z* 607.4220 (calcd for C_36_H_55_N_4_O_4_, 607.4218).

#### 2.2.14. *N*^1^,*N*^12^-Bis(3-((*E*)-3-(4-methoxyphenyl)acrylamido)propyl)dodecane-1,12-diaminium 2,2,2-trifluoroacetate (**14f**)

Using general procedure B, reaction of 4-methoxycinnamic acid (**6**) (46 mg, 0.258 mmol), EDC·HCl (55 mg, 0.287 mmol), DMAP (63 mg, 0.516 mmol) and di-*tert*-butyl dodecane-1,12-diylbis((3-aminopropyl)carbamate) (**12f**) (50 mg, 0.097 mmol) afforded di-*tert*-butyl dodecane-1,12-diylbis((3-((*E*)-3-(4-methoxyphenyl)acrylamido)propyl)carbamate) as a white oil (37 mg, 46%). Using general procedure C, a sub-sample of this product (19 mg, 0.023 mmol) was deprotected to afford the di-TFA salt **14f** as a yellow oil (12 mg, 61%). R*_f_* = 0.09 (MeOH:10% HCl, 7:3); IR (ATR) ν_max_ 2930, 1673, 1603, 1513, 1202, 1175, 1133, 831, 721 cm^−1^; ^1^H NMR (DMSO-*d*_6_, 400 MHz) δ 8.48 (4H, br s, NH_2_-5′), 8.26 (2H, t, *J* = 5.7 Hz, NH-1′), 7.51 (4H, d, *J* = 8.7 Hz, 2H-5), 7.38 (2H, d, *J =* 15.7 Hz, H-3), 6.97 (4H, d, *J* = 8.7 Hz, 2H-6), 6.48 (2H, d, *J* = 15.7 Hz, H-2), 3.78 (6H, s, OMe), 3.25 (4H, dt, *J* = 6.4, 6.4 Hz, H_2_-2′), 2.95–2.84 (8H, m, H_2_-4′, H_2_-6′), 1.78 (4H, tt, *J* = 7.5, 7.5 Hz, H_2_-3′), 1.60–1.52 (4H, m, H_2_-7′), 1.31–1.22 (16H, m, H_2_-8′, H_2_-9′, H_2_-10′, H_2_-11′); ^13^C NMR (DMSO-*d*_6_, 100 MHz) δ 165.8 (C-1), 160.4 (C-7), 138.6 (C-3), 129.1 (C-5), 127.3 (C-4), 119.3 (C-2), 114.4 (C-6), 55.3 (OMe), 46.8 (C-6′), 44.7 (C-4′), 35.8 (C-2′), 28.9 (C-9′/C-10′/C-11′), 28.8 (C-9′/C-10′/C-11′), 28.5 (C-9′/C-10′/C-11′), 26.2 (C-3′), 25.9 (C-7′/C-8′), 25.5 (C-7′/C-8′); (+)-HRESIMS [M+H]^+^ *m*/*z* 635.4532 (calcd for C_38_H_59_N_4_O_4_, 635.4531).

#### 2.2.15. *N*^1^,*N*^4^-Bis(3-((*E*)-3-(3-bromo-4-methoxyphenyl)acrylamido)propyl)butane-1,4-diaminium 2,2,2-trifluoroacetate (**15a**)

Using general procedure B, reaction of 3-bromo-4-methoxycinnamic acid (**7**) (50 mg, 0.194 mmol), EDC·HCl (42 mg, 0.219 mmol), DMAP (48 mg, 0.39 mmol) and di-*tert*-butyl butane 1,4-diylbis((3-aminopropyl)carbamate) (**12a**) (50 mg, 0.124 mmol) afforded di-*tert*-butyl butane-1,4-diylbis((3-((*E*)-3-(3-bromo-4-methoxyphenyl)acrylamido) propyl)carbamate) as a white oil (50 mg, 36%). Using general procedure C, a sub-sample of this product (25 mg, 0.028 mmol) was deprotected to afford the di-TFA salt **15a** as a yellow oil (7 mg, 27%). R*_f_* = 0.29 (MeOH:10% HCl, 7:3); IR (ATR) ν_max_ 3284, 2843, 1673, 1497, 1261, 1201, 1131, 1052, 800, 722 cm^−1^; ^1^H NMR (DMSO-*d*_6_, 400 MHz) δ 8.57 (4H, br s, NH_2_-5′), 8.25 (2H, t, *J* = 5.7 Hz, NH-1′), 7.80 (2H, d, *J* = 2.1 Hz, H-5), 7.57 (2H, dd, *J* = 8.6, 2.1 Hz, H-9), 7.36 (2H, d, *J* = 16.0 Hz, H-3), 7.15 (2H, d, *J* = 8.6 Hz, H-8), 6.54 (2H, d, *J* = 16.0 Hz, H-2), 3.88 (6H, s, OMe), 3.25 (4H, dt, *J* = 6.4, 6.4 Hz, H_2_-2′), 2.97–2.90 (8H, m, H_2_-4′, H_2_-6′), 1.79 (4H, tt, *J* = 7.5, 7.5 Hz, H_2_-3′), 1.65–1.61 (4H, m, H_2_-7′); ^13^C NMR (DMSO-*d*_6_, 100 MHz) δ 165.4 (C-1), 156.3 (C-7), 137.2 (C-3), 131.7 (C-5), 128.9 (C-4), 128.5 (C-9), 120.9 (C-2), 112.9 (C-8), 111.1 (C-6), 56.4 (OMe), 46.1 (C-6′), 44.7 (C-4′), 35.9 (C-2′), 26.1 (C-3′), 22.7 (C-7′); (+)-HRESIMS [M+H]^+^ *m*/*z* 679.1476 (calcd for C_30_H_41_^79^Br_2_N_4_O_4_, 679.1489).

#### 2.2.16. *N*^1^,*N*^6^-Bis(3-((*E*)-3-(3-bromo-4-methoxyphenyl)acrylamido)propyl)hexane-1,6-diaminium 2,2,2-trifluoroacetate (**15b**)

Using general procedure B, reaction of 3-bromo-4-methoxycinnamic acid (**7**) (50 mg, 0.194 mmol), EDC·HCl (42 mg, 0.219 mmol), DMAP (47 mg, 0.385 mmol) and di-*tert*-butyl hexane-1,6-diylbis((3-aminopropyl)carbamate) (**12b**) (34 mg, 0.079 mmol) afforded di-*tert*-butyl hexane-1,6-diylbis((3-((*E*)-3-(3-bromo-4-methoxyphenyl)acrylamido) propyl) carbamate as a white oil (42 mg, 58%). Using general procedure C, a sub-sample of this product (21 mg, 0.023 mmol) was deprotected to afford the di-TFA salt **15b** as a yellow oil (10 mg, 46%). R*_f_* = 0.29 (MeOH:10% HCl, 7:3); IR (ATR) ν_max_ 3285, 2945, 2845, 1659, 1596, 1497, 1261, 1200, 1133, 1052, 801, 722 cm^−1^; ^1^H NMR (DMSO-*d*_6_, 400 MHz) δ 8.51 (4H, br s, NH_2_-5′), 8.25 (2H, t, *J* = 5.6 Hz, NH-1′), 7.80 (2H, d, *J* = 2.1 Hz, H-5), 7.57 (2H, dd, *J* = 8.5, 2.1 Hz, H-9), 7.36 (2H, d, *J* = 15.9 Hz, H-3), 7.15 (2H, d, *J* = 8.5 Hz, H-8), 6.54 (2H, d, *J* = 15.7 Hz, H-2), 3.88 (6H, s, OMe), 3.25 (4H, dt, *J* = 6.4, 6.4 Hz, H_2_-2′), 2.95–2.86 (8H, m, H_2_-4′, H_2_-6′), 1.79 (4H, tt, *J* = 7.5, 7.5 Hz, H_2_-3′), 1.60–1.54 (4H, m H_2_-7′), 1.34–1.29 (4H, m, H_2_-8′); ^13^C NMR (DMSO-*d*_6_, 100 MHz) δ 165.4 (C-1), 156.2 (C-7), 137.1 (C-3), 131.7 (C-5), 128.9 (C-4), 128.5 (C-9), 120.9 (C-2), 112.9 (C-8), 111.1 (C-6), 56.4 (OMe), 46.6 (C-6′), 44.7 (C-4′), 35.9 (C-2′), 26.1 (C-3′), 25.5 (C-7′/C-8′), 25.4 (C-7′/C-8′); (+)-HRESIMS [M+H]^+^ *m*/*z* 707.1782 (calcd for C_32_H_45_^79^Br_2_N_4_O_4_, 707.1802).

#### 2.2.17. *N*^1^,*N*^7^-Bis(3-((*E*)-3-(3-bromo-4-methoxyphenyl)acrylamido)propyl)heptane-1,7-diaminium 2,2,2-trifluoroacetate (**15c**)

Using general procedure B, reaction of 3-bromo-4-methoxycinnamic acid (**7**) (77 mg, 0.300 mmol), EDC·HCl (65 mg, 0.339 mmol), DMAP (73 mg, 0.600 mmol) and di-*tert*-butyl heptane-1,7-diylbis((3-aminopropyl)carbamate) (**12c**) (50 mg, 0.11 mmol) afforded di-*tert*-butyl heptane-1,7-diylbis((3-((*E*)-3-(3-bromo-4-methoxyphenyl)acrylamido) propyl)carbamate) as a white oil (61 mg, 59%). Using general procedure C, a sub-sample of this product (31 mg, 0.034 mmol) was deprotected to afford the di-TFA salt **15c** as a yellow oil (22 mg, 69%). R*_f_* = 0.20 (MeOH:10% HCl, 7:3); IR (ATR) ν_max_ 2971, 1738, 1676, 1498, 1366, 1204, 1134, 1053, 800, 722 cm^−1^; ^1^H NMR (DMSO-*d*_6_, 400 MHz) δ 8.53 (4H, br s, NH_2_-5′), 8.25 (2H, t, *J* = 5.9 Hz, NH-1′), 7.80 (2H, d, *J* = 2.2 Hz, H-5), 7.57 (2H, dd, *J* = 8.5, 2.0 Hz, H-9), 7.36 (2H, d, *J* = 15.7 Hz, H-3), 7.15 (2H, d, *J* = 8.7 Hz, H-8), 6.55 (2H, d, *J* = 15.9 Hz, H-2), 3.88 (6H, s, OMe), 3.25 (4H, dt, *J* = 6.4, 6.4 Hz, H_2_-2′), 2.95–2.86 (8H, m, H_2_-4′, H_2_-6′), 1.79 (4H, tt, *J* = 7.5, 7.5 Hz, H_2_-3′), 1.60–1.53 (4H, m, H_2_-7′), 1.32–1.27 (6H, m, H_2_-8′, H_2_-9′); ^13^C NMR (DMSO-*d*_6_, 100 MHz) δ 165.4 (C-1), 156.2 (C-7), 137.1 (C-3), 131.7 (C-5), 129.0 (C-4), 128.5 (C-9), 120.9 (C-2), 112.9 (C-8), 111.1 (C-6), 56.4 (OMe), 46.7 (C-6′), 44.7 (C-4′), 35.9 (C-2′), 28.0 (C-8′/C-9′), 26.1 (C-3′), 25.7 (C-8′/C-9′), 25.4 (C-7′); (+)-HRESIMS [M+H]^+^ *m*/*z* 721.1961 (calcd for C_33_H_47_^79^Br_2_N_4_O_4_, 721.1959).

#### 2.2.18. *N*^1^,*N*^8^-Bis(3-((*E*)-3-(3-bromo-4-methoxyphenyl)acrylamido)propyl)octane-1,8-diaminium 2,2,2-trifluoroacetate (**15d**)

Using general procedure B, reaction of 3-bromo-4-methoxycinnamic acid (**7**) (75 mg, 0.29 mmol), EDC·HCl (62 mg, 0.323 mmol), DMAP (71 mg, 0.58 mmol) and di-*tert*-butyl octane-1,8-diylbis((3-aminopropyl)carbamate) (**12d**) (50 mg, 0.109 mmol) afforded di-*tert*-butyl octane-1,8-diylbis((3-((*E*)-3-(3-bromo-4-methoxyphenyl)acrylamido) propyl)carbamate) as a white oil (79 mg, 77%). Using general procedure C, a sub-sample of this product (40 mg, 0.043 mmol) was deprotected to afford the di-TFA salt **15d** as a yellow oil (29 mg, 70%). R*_f_* = 0.23 (MeOH:10% HCl, 7:3); IR (ATR) ν_max_ 3395, 1678, 1498, 1202, 1133, 1025, 998, 827, 721 cm^−1^; ^1^H NMR (DMSO-*d*_6_, 400 MHz) δ 8.60 (4H, br s, NH_2_-5′), 8.28 (2H, t, *J* = 5.8 Hz, NH-1′), 7.80 (2H, d, *J* = 2.3 Hz, H-5), 7.57 (2H, dd, *J* = 8.8, 2.1 Hz, H-9), 7.35 (2H, d, *J* = 15.7 Hz, H-3), 7.15 (2H, d, *J* = 8.9 Hz, H-8), 6.55 (2H, d, *J* = 15.7 Hz, H-2), 3.88 (6H, s, OMe), 3.25 (4H, dt, *J* = 6.4, 6.4 Hz, H_2_-2′), 2.95–2.85 (8H, m, H_2_-4′, H_2_-6′), 1.79 (4H, tt, *J* = 7.5, 7.5 Hz, H_2_-3′), 1.60–1.53 (4H, m, H_2_-7′), 1.32–1.23 (8H, m, H_2_-8′, H_2_-9′); ^13^C NMR (DMSO-*d*_6_, 100 MHz) δ 165.4 (C-1), 156.2 (C-7), 137.1 (C-3), 131.8 (C-5), 129.0 (C-4), 128.5 (C-9), 121.0 (C-2), 112.9 (C-8), 111.1 (C-6), 56.4 (OMe), 46.8 (C-6′), 44.7 (C-4′), 35.9 (C-2′), 28.3 (C-8′/C-9′), 26.1 (C-3′), 25.8 (C-8′/C-9′), 25.4 (C-7′); (+)-HRESIMS [M+H]^+^ *m*/*z* 735.2115 (calcd for C_34_H_49_^79^Br_2_N_4_O_4_, 735.2115).

#### 2.2.19. *N*^1^,*N*^10^-Bis(3-((*E*)-3-(3-bromo-4-methoxyphenyl)acrylamido)propyl)decane-1,10-diaminium 2,2,2-trifluoroacetate (**15e**)

Using general procedure B, reaction of 3-bromo-4-methoxycinnamic acid (**7**) (70 mg, 0.273 mmol), EDC·HCl (59 mg, 0.308 mmol), DMAP (67 mg, 0.548 mmol) and di-*tert*-butyl decane-1,10-diylbis((3-aminopropyl)carbamate) (**12e**) (50 mg, 0.103 mmol) afforded di-*tert*-butyl decane-1,10-diylbis((3-((*E*)-3-(3-bromo-4-methoxyphenyl)acrylamido) propyl)carbamate) as a white oil (87 mg, 88%). Using general procedure C, a sub-sample of this product (25 mg, 0.026 mmol) was deprotected to afford the di-TFA salt **15e** as a yellow oil (10 mg, 39%). R*_f_* = 0.11 (MeOH:10% HCl, 7:3); IR (ATR) ν_max_ 2931, 2854, 1672, 1596, 1498, 1261, 1201, 1053, 800, 721 cm^−1^; ^1^H NMR (DMSO-*d*_6_, 400 MHz) δ 8.37 (4H, br s, NH_2_-5′), 8.21 (2H, t, *J* = 5.7 Hz, NH-1′), 7.80 (2H, d, *J* = 2.3 Hz, H-5), 7.57 (2H, dd, *J* = 8.2, 2.1 Hz, H-9), 7.36 (2H, d, *J* = 15.8 Hz, H-3), 7.16 (2H, d, *J* = 8.2 Hz, H-8), 6.53 (2H, d, *J* = 15.8 Hz, H-2), 3.88 (6H, s, OMe), 3.25 (4H, dt, *J* = 6.4, 6.4 Hz, H_2_-2′), 2.94–2.86 (8H, m, H_2_-4′, H_2_-6′), 1.78 (4H, tt, *J* = 7.5, 7.5 Hz, H_2_-3′), 1.58–1.52 (4H, m, H_2_-7′), 1.32–1.23 (12H, m, H_2_-8′, H_2_-9′, H_2_-10′); ^13^C NMR (DMSO-*d*_6_, 100 MHz) δ 165.5 (C-1), 156.3 (C-7), 137.3 (C-3), 131.8 (C-5), 129.0 (C-4), 128.6 (C-9), 120.9 (C-2), 112.9 (C-8), 111.2 (C-6), 56.5 (OMe), 46.8 (C-6′), 44.7 (C-4′), 35.9 (C-2′), 28.8 (C-9′/C-10′), 28.6 (C-9′/C-10′), 26.2 (C-3′), 25.9 (C-8′), 25.5 (C-7′); (+)-HRESIMS [M+H]^+^ *m*/*z* 763.2427 (calcd for C_36_H_53_^79^Br_2_N_4_O_4_, 763.2428).

#### 2.2.20. *N*^1^,*N*^12^-Bis(3-((*E*)-3-(3-bromo-4-methoxyphenyl)acrylamido)propyl)dodecane-1,12-diaminium 2,2,2-trifluoroacetate (**15f**)

Using general procedure B, reaction of 3-bromo-4-methoxycinnamic acid (**7**) (66 mg, 0.257 mmol), EDC·HCl (55 mg, 0.287 mmol), DMAP (63 mg, 0.516 mmol) and di-*tert*-butyl dodecane-1,12-diylbis((3-aminopropyl)carbamate) (**12f**) (50 mg, 0.097 mmol) afforded di-*tert*-butyl dodecane-1,12-diylbis ((3-((*E*)-3-(3-bromo-4-methoxyphenyl)acrylamido)propyl)carbamate) as a white oil (41 mg, 43%). Using general procedure C, a sub-sample of this product (21 mg, 0.021 mmol) was deprotected to afford the di-TFA salt **15f** as a yellow oil (6 mg, 28%). R*_f_* = 0.09 (MeOH:10% HCl, 7:3); IR (ATR) ν_max_ 2930, 1676, 1498, 1262, 1202, 1134, 1053, 800, 722 cm^−1^; ^1^H NMR (DMSO-*d*_6_, 400 MHz) δ 8.44 (4H, br s, NH_2_-5′), 8.24 (2H, t, *J* = 5.4 Hz, NH-1′), 7.80 (2H, d, *J* = 2.3 Hz, H-5), 7.57 (2H, dd, *J* = 8.6, 2.1 Hz, H-9), 7.36 (2H, d, *J* = 15.8 Hz, H-3), 7.15 (2H, d, *J* = 8.5 Hz, H-8), 6.54 (2H, d, *J* = 15.8 Hz, H-2), 3.88 (6H, s, OMe), 3.25 (4H, dt, *J* = 6.4, 6.4 Hz, H_2_-2′), 2.91–2.84 (8H, m, H_2_-4′, H_2_-6′), 1.78 (4H, tt, *J* = 7.5, 7.5 Hz, H_2_-3′), 1.59–1.52 (4H, m, H_2_-7′), 1.30–1.22 (16H, m, H_2_-8′, H_2_-9′, H_2_-10′, H_2_-11′); ^13^C NMR (DMSO-*d*_6_, 100 MHz) δ 165.5 (C-1), 156.3 (C-7), 137.2 (C-3), 131.7 (C-5), 129.0 (C-4), 128.5 (C-9), 120.9 (C-2), 112.9 (C-8), 111.1 (C-6), 56.4 (OMe), 46.8 (C-6′), 44.7 (C-4′), 35.9 (C-2′), 29.0 (C-9′/C-10′/C-11′), 28.9 (C-9′/C-10′/C-11′), 28.5 (C-9′/C-10′/C-11′), 26.1 (C-3′), 25.9 (C-8′), 25.5 (C-7′); (+)-HRESIMS [M+H]^+^ *m*/*z* 791.2710 (calcd for C_38_H_57_^79^Br_2_N_4_O_4_, 791.2741).

#### 2.2.21. *N*^1^,*N*^4^-Bis(3-((*E*)-3-(3,5-dibromo-4-methoxyphenyl)acrylamido)propyl)butane-1,4-diaminium 2,2,2-trifluoroacetate (**16a**)

Using general procedure B, reaction of 3,5-dibromo-4-methoxycinnamic acid (**8**) (109 mg, 0.324 mmol), EDC·HCl (67 mg, 0.350 mmol), DMAP (76 mg, 0.622 mmol) and di-*tert*-butyl butane-1,4-diylbis((3-aminopropyl)carbamate) (**12a**) (50 mg, 0.124 mmol) afforded di-*tert*-butyl butane-1,4-diylbis((3-((*E*)-3-(3,5-dibromo-4-methoxyphenyl)acrylamido)propyl)carbamate) as a white oil (61 mg, 47%). Using general procedure C, a sub-sample of this product (31 mg, 0.030 mmol) was deprotected to afford the di-TFA salt **16a** as a yellow oil (19 mg, 60%). R*_f_* = 0.08 (MeOH:10% HCl, 7:3); IR (ATR) ν_max_ 1738, 1366, 1217, 733 cm^−1^; ^1^H NMR (DMSO-*d*_6_, 400 MHz) δ 8.61 (4H, br s, NH_2_-5′), 8.30 (2H, t, *J* = 5.8 Hz, NH-1′), 7.88 (4H, s, 2H-5), 7.35 (2H, d, *J =* 15.9 Hz, H-3), 6.66 (2H, d, *J* = 15.9 Hz, H-2), 3.81 (6H, s, OMe), 3.25 (4H, dt, *J* = 6.4, 6.4 Hz, H_2_-2′), 2.96–2.90 (8H, m, H_2_-4′, H_2_-6′), 1.79 (4H, tt, *J* = 7.5, 7.5 Hz, H_2_-3′), 1.65–1.61 (4H, m, H_2_-7′); ^13^C NMR (DMSO-*d*_6_, 100 MHz) δ 164.9 (C-1), 154.0 (C-7), 135.5 (C-3), 134.3 (C-4), 131.6 (C-5), 124.1 (C-2), 118.0 (C-6), 60.6 (OMe), 46.1 (C-6′), 44.7 (C-4′), 36.0 (C-2′), 26.0 (C-3′), 22.7 (C-7′); (+)-HRESIMS [M+H]^+^ *m*/*z* 834.9688 (calcd for C_30_H_39_^79^Br_4_N_4_O_4_, 834.9699). The ^1^H and ^13^C NMR data agreed with those reported in the literature [19].

#### 2.2.22. *N*^1^,*N*^6^-Bis(3-((*E*)-3-(3,5-dibromo-4-methoxyphenyl)acrylamido)propyl)hexane-1,6-diaminium 2,2,2-trifluoroacetate (**16b**)

Following general procedure B, reaction of 3,5-dibromo-4-methoxycinnamic acid (**8**) (102 mg, 0.304 mmol), EDC·HCl (62 mg, 0.323 mmol), DMAP (71 mg, 0.581 mmol) and di-*tert*-butyl hexane-1,6-diylbis((3-aminopropyl)carbamate) (**12b**) (50 mg, 0.116 mmol) afforded di-*tert*-butylhexane-1,6-diylbis((3-((*E*)-3-(3,5-dibromo-4-methoxyphenyl)acrylamido)propyl)carbamate) as a white oil (72 mg, 58%). Using general procedure C, a sub-sample of this product (36 mg, 0.034 mmol) was deprotected to afford the di-TFA salt **16b** as a yellow oil (23 mg, 62%). R*_f_* = 0.08 (MeOH:10% HCl, 7:3); IR (ATR) ν_max_ 2971, 1738, 1366, 1217, 733 cm^−1^; ^1^H NMR (DMSO-*d*_6_, 400 MHz) δ 8.50 (4H, br s, NH_2_-5′), 8.29 (2H, t, *J* = 5.8 Hz, NH-1′), 7.88 (4H, s, 2H-5), 7.35 (2H, d, *J =* 15.6 Hz, H-3), 6.66 (2H, d, *J* = 15.6 Hz, H-2), 3.82 (6H, s, OMe), 3.25 (4H, dt, *J* = 6.4, 6.4 Hz, H_2_-2′), 2.95–1.85 (8H, m, H_2_-4′, H_2_-6′), 1.79 (4H, tt, *J* = 7.5, 7.5 Hz, H_2_-3′), 1.60–1.53 (4H, m, H_2_-7′), 1.34–1.29 (4H, m, H_2_-8′); ^13^C NMR (DMSO-*d*_6_, 100 MHz) δ 164.9 (C-1), 153.9 (C-7), 135.4 (C-3), 134.3 (C-4), 131.6 (C-5), 124.2 (C-2), 118.0 (C-6), 60.6 (OMe), 46.7 (C-6′), 44.7 (C-4′), 36.0 (C-2′), 26.0 (C-3′), 25.5 (C-7′/C-8′), 25.4 (C-7′/C-8′); (+)-HRESIMS [M+H]^+^ *m*/*z* 862.9996 (calcd for C_32_H_43_^79^Br_4_N_4_O_4_, 863.0012). The ^1^H and ^13^C NMR data agreed with those reported in the literature [19]. The literature data reported for this compound contains an extra, spurious, ^13^C NMR signal at δ_C_ 115.2.

#### 2.2.23. *N*^1^,*N*^7^-Bis(3-((*E*)-3-(3,5-dibromo-4-methoxyphenyl)acrylamido)propyl)heptane-1,7-diaminium 2,2,2-trifluoroacetate (**16c**)

Following general procedure B, reaction of 3,5-dibromo-4-methoxycinnamic acid (**8**) (98 mg, 0.292 mmol), EDC·HCl (60 mg, 0.313 mmol), DMAP (69 mg, 0.565 mmol) and di-*tert*-butyl heptane-1,7-diylbis((3-aminopropyl)carbamate) (**12c**) (50 mg, 0.112 mmol) afforded di-*tert*-butylheptane-1,7-diylbis((3-((*E*)-3-(3,5-dibromo-4-methoxyphenyl)acrylamido)propyl)carbamate) as a white oil (71 mg, 58%). Using general procedure C, a sub-sample of this product (36 mg, 0.033 mmol) was deprotected to afford the di-TFA salt **16c** as a yellow oil (22 mg, 60%). R*_f_* = 0.08 (MeOH:10% HCl, 7:3); IR (ATR) ν_max_ 2938, 1665, 1473, 1202, 1133, 984, 800, 721 cm^−1^; ^1^H NMR (DMSO-*d*_6_, 400 MHz) δ 8.45 (4H, br s, NH_2_-5′), 8.27 (2H, t, *J* = 5.9 Hz, NH-1′), 7.89 (4H, s, 2H-5), 7.35 (2H, d, *J =* 16.0 Hz, H-3), 6.66 (2H, d, *J* = 16.0 Hz, H-2), 3.82 (6H, s, OMe), 3.25 (4H, dt, *J* = 6.4, 6.4 Hz, H_2_-2′), 2.95–2.85 (8H, m, H_2_-4′, H_2_-6′), 1.79 (4H, tt, *J* = 7.5, 7.5 Hz, H_2_-3′), 1.59–1.53 (4H, m, H_2_-7′), 1.31–1.27 (6H, m, H_2_-8′, H_2_-9′); ^13^C NMR (DMSO-*d*_6_, 100 MHz) δ 164.9 (C-1), 154.0 (C-7), 135.5 (C-3), 134.3 (C-4), 131.6 (C-5), 124.1 (C-2), 118.0 (C-6), 60.6 (OMe), 46.7 (C-6′), 44.7 (C-4′), 36.0 (C-2′), 28.0 (C-9′), 26.0 (C-3′), 25.8 (C-7′/C-8′), 25.4 (C-7′/C-8′); (+)-HRESIMS [M+H]^+^ *m*/*z* 877.0168 (calcd for C_33_H_45_^79^Br_4_N_4_O_4_, 877.0169).

#### 2.2.24. *N*^1^,*N*^8^-Bis(3-((*E*)-3-(3,5-dibromo-4-methoxyphenyl)acrylamido)propyl)octane-1,8-diaminium 2,2,2-trifluoroacetate (**16d**)

Following general procedure B, reaction of 3,5-dibromo-4-methoxycinnamic acid (**8**) (95 mg, 0.283 mmol), EDC·HCl (59 mg, 0.308 mmol), DMAP (66 mg, 0.540 mmol) and di-*tert*-butyl octane-1,8-diylbis((3-aminopropyl)carbamate) (**12d**) (50 mg, 0.109 mmol) afforded di-*tert*-butyloctane-1,8-diylbis((3-((*E*)-3-(3,5-dibromo-4-methoxyphenyl)acrylamido) propyl)carbamate) as a white oil (57 mg, 48%). Using general procedure C, a sub-sample of this product (28.5 mg, 0.026 mmol) was deprotected to afford the di-TFA salt **16d** as a yellow oil (22 mg, 75%). R*_f_* = 0.08 (MeOH:10% HCl, 7:3); IR (ATR) ν_max_ 1740, 1366, 1217 cm^−1^; ^1^H NMR (DMSO-*d*_6_, 400 MHz) δ 8.46 (4H, br s, NH_2_-5′), 8.27 (2H, t, *J* = 5.8 Hz, NH-1′), 7.88 (4H, s, 2H-5), 7.36 (2H, d, *J =* 16.0 Hz, H-3), 6.66 (2H, d, *J* = 16.0 Hz, H-2), 3.81 (6H, s, OMe), 3.25 (4H, dt, *J* = 6.4, 6.4 Hz, H_2_-2′), 2.94–2.85 (8H, m, H_2_-4′, H_2_-6′), 1.78 (4H, tt, *J* = 7.5, 7.5 Hz, H_2_-3′), 1.59–1.52 (4H, m, H_2_-7′), 1.31–1.25 (8H, m, H_2_-8′, H_2_-9′); ^13^C NMR (DMSO-*d*_6_, 100 MHz) δ 164.9 (C-1), 154.0 (C-7), 135.5 (C-3), 134.3 (C-4), 131.6 (C-5), 124.2 (C-2), 118.0 (C-6), 60.6 (OMe), 46.8 (C-6′), 44.7 (C-4′), 36.0 (C-2′), 28.3 (C-8′/C-9′), 26.0 (C-3′), 25.8 (C-8′/C-9′), 25.5 (C-7′); (+)-HRESIMS [M+H]^+^ *m*/*z* 891.0316 (calcd for C_34_H_47_^79^Br_4_N_4_O_4_, 891.0325).

#### 2.2.25. *N*^1^,*N*^10^-Bis(3-((*E*)-3-(3,5-dibromo-4-methoxyphenyl)acrylamido)propyl)decane-1,10-diaminium 2,2,2-trifluoroacetate (**16e**)

Using general procedure B, reaction of 3,5-dibromo-4-methoxycinnamic acid (**8**) (90 mg, 0.268 mmol), EDC·HCl (55 mg, 0.287 mmol), DMAP (63 mg, 0.516 mmol) and di-*tert*-butyl decane-1,10-diylbis((3-aminopropyl)carbamate) (**12e**) (50 mg, 0.103 mmol) afforded di-*tert*-butyldecane-1,10-diylbis((3-((*E*)-3-(3,5-dibromo-4-methox-phenyl)acrylamido)propyl)carbamate) as a white oil (30 mg, 26%). Using general procedure C, a sub-sample of this product (22 mg, 0.020 mmol) was deprotected to afford the di-TFA salt **16e** as a yellow oil (18 mg, 80%). R*_f_* = 0.08 (MeOH:10% HCl, 7:3); IR (ATR) ν_max_ 2933, 1674, 1473, 1203, 1135, 800, 722 cm^−1^; ^1^H NMR (DMSO-*d*_6_, 400 MHz) δ 8.46 (4H, br s, NH_2_-5′), 8.28 (2H, t, *J* = 5.8 Hz, NH-1′), 7.88 (4H, s, 2H-5), 7.35 (2H, d, *J =* 15.7 Hz, H-3), 6.66 (2H, d, *J* = 15.7 Hz, H-2), 3.82 (6H, s, OMe), 3.25 (4H, dt, *J* = 6.4, 6.4 Hz, H_2_-2′), 2.94–2.85 (8H, m, H_2_-4′, H_2_-6′), 1.78 (4H, tt, *J* = 7.5, 7.5 Hz, H_2_-3′), 1.59–1.52 (4H, m, H_2_-7′), 1.30–1.23 (12H, m, H_2_-8′, H_2_-9′, H_2_-10′); ^13^C NMR (DMSO-*d*_6_, 100 MHz) δ 164.9 (C-1), 153.9 (C-7), 135.4 (C-3), 134.3 (C-4), 131.6 (C-5), 124.1 (C-2), 118.0 (C-6), 60.5 (OMe), 46.8 (C-6′), 44.6 (C-4′), 35.9 (C-2′), 28.7 (C-9′/C-10′), 28.5 (C-9′/C-10′), 26.0 (C-3′), 25.9 (C-8′), 25.5 (C-7′); (+)-HRESIMS [M+H]^+^ *m*/*z* 919.0649 (calcd for C_36_H_51_^79^Br_4_N_4_O_4_, 919.0638).

#### 2.2.26. *N*^1^,*N*^12^-Bis(3-((*E*)-3-(3,5-dibromo-4-methoxyphenyl)acrylamido)propyl)dodecane-1,12-diaminium 2,2,2-trifluoroacetate (**16f**)

Using general procedure B, reaction of 3,5-dibromo-4-methoxycinnamic acid (**8**) (85 mg, 0.253 mmol), EDC·HCl (52 mg, 0.271 mmol), DMAP (59 mg, 0.483 mmol) and di-*tert*-butyl dodecane-1,12-diylbis((3-aminopropyl)carbamate) (**12f**) (50 mg, 0.097 mmol) afforded di-*tert*-butyldodecane-1,12-diylbis((3-((*E*)-3-(3,5-dibromo-4-methoxyphenyl)acrylamido)propyl)carbamate) as a white oil (44 mg, 39%). Using general procedure C, a sub-sample of this product (22 mg, 0.019 mmol) was deprotected to afford the di-TFA salt **16f** as a yellow oil (16 mg, 71%). R*_f_* = 0.08 (MeOH:10% HCl, 7:3); IR (ATR) ν_max_ 2931, 1675, 1366, 1203, 722 cm^−1^; ^1^H NMR (DMSO-*d*_6_, 400 MHz) δ 8.42 (4H, br s, NH_2_-5′), 8.27 (2H, t, *J* = 5.8 Hz, NH-1′), 7.88 (4H, s, 2H-5), 7.35 (2H, d, *J =* 15.6 Hz, H-3), 6.66 (2H, d, *J* = 15.6 Hz, H-2), 3.82 (6H, s, OMe), 3.25 (4H, dt, *J* = 6.4, 6.4 Hz, H_2_-2′), 2.94–2.84 (8H, m, H_2_-4′, H_2_-6′), 1.78 (4H, tt, *J* = 7.5, 7.5 Hz, H_2_-3′), 1.58–1.51 (4H, m, H_2_-7′), 1.30–1.22 (16H, m, H_2_-8′, H_2_-9′, H_2_-10′, H_2_-11′); ^13^C NMR (DMSO-*d*_6_, 100 MHz) δ 164.9 (C-1), 153.9 (C-7), 135.5 (C-3), 134.3 (C-4), 131.6 (C-5), 124.1 (C-2), 118.0 (C-6), 60.5 (OMe), 46.8 (C-6′), 44.6 (C-4′), 35.9 (C-2′), 29.0 (C-9′/C-10′/C-11′), 28.9 (C-9′/C-10′/C-11′), 28.5 (C-9′/C-10′/C-11′), 26.0 (C-3′), 25.9 (C-8′), 25.5 (C-7′); (+)-HRESIMS [M+H]^+^ *m*/*z* 947.0951 (calcd for C_38_H_55_^79^Br_4_N_4_O_4_, 947.0951).

### 2.3. Antimicrobial Assays

The susceptibility of bacterial strains *S. aureus* (ATCC 25923), *E. coli* (ATCC 25922) and *P. aeruginosa* (ATCC 27853) to antibiotics and compounds was determined using protocols reported previously [28]. Additional antimicrobial evaluation against MRSA (ATCC 43300), *Klebsiella pneumoniae* (ATCC 700603), *Acinetobacter baumannii* (ATCC 19606), *Candida albicans* (ATCC 90028) and *Cryptococcus neoformans* (ATCC 208821) was undertaken at the Community for Open Antimicrobial Drug Discovery at The University of Queensland (Australia) according to their standard protocols reported previously [28,29].

### 2.4. Determination of the MICs of Antibiotics in the Presence of Synergizing Compounds

Antibiotic enhancer concentrations were determined using previously reported protocols [28].

### 2.5. Cytotoxicity Assays

Cytotoxicity assays were conducted using the protocols previously reported [28].

### 2.6. Hemolytic Assay

Hemolysis assays were conducted using the protocols previously reported [28].

## 3. Results and Discussion

**Figure 2:**
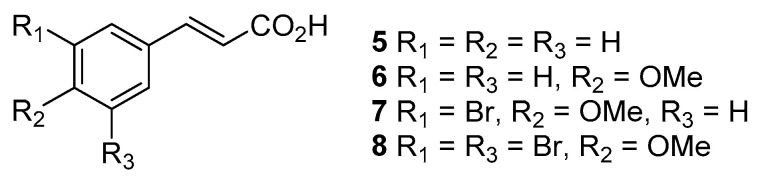
Structures of cinnamic acid head groups **5**–**8**.

To explore variation of the extent of substitution on the aromatic end-group of ianthelliformisamine C, we made use of a set of four cinnamic acids including cinnamic acid (**5**), 4-methoxycinnamic acid (**6**), 3-bromo-4-methoxycinnamic acid (**7**) and 3,5-dibromo-4-methoxycinnamic acid (**8**) (Figure 2).

**Scheme 1:**
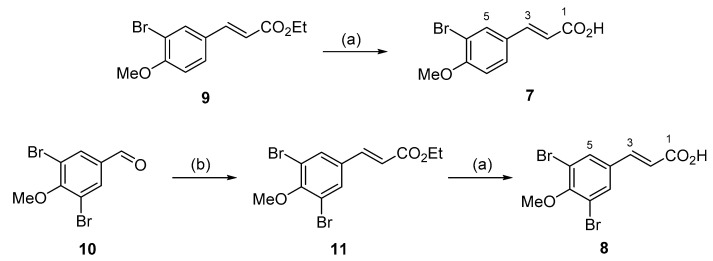
Synthetic routes for the preparation of cinnamic acids **7** and **8**. Reagents and conditions: (a) 1N NaOH, EtOH, reflux, N_2_, 4 h, 87% for **7**; 86% for **8**; (b) NaH, triethyl phosphonoacetate, rt, N_2_, 24 h, 68%.

Of these four cinnamic acids, **7** and **8** were not commercially available, requiring synthesis. (*E*)-3-(3-Bromo-4-methoxyphenyl)acrylic acid (**7**) was prepared by saponification of the previously reported ethyl cinnamate **9** [22] in 87% yield (Scheme 1). The corresponding dibromo-cinnamic acid **8** was prepared by a similar sequence as reported previously by Khan et al. [19], making use of slightly different procedures. Wittig olefination of aldehyde **10** with triethyl phosphonoacetate and NaH afforded ethyl ester **11** in 68% yield, saponification of which, using aq. NaOH in EtOH, afforded cinnamic acid **8** in 86% yield.

**Figure 3:**
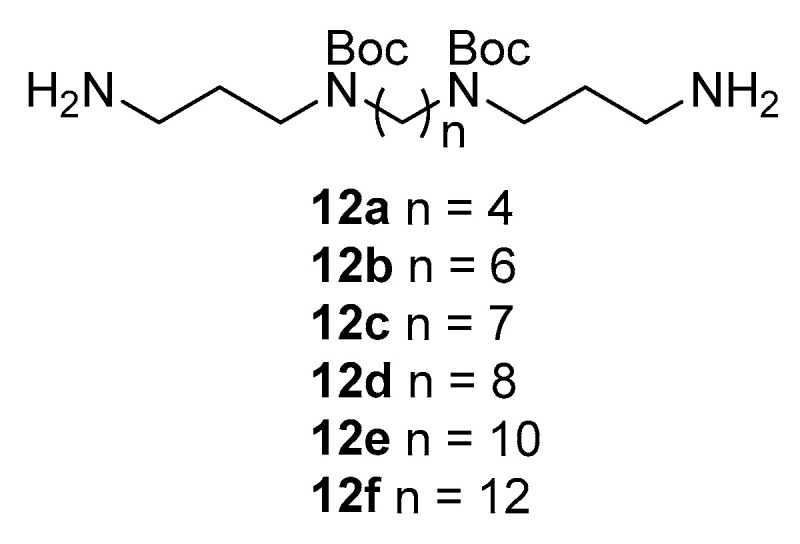
Boc-protected polyamines **12a**–**f**.

Six polyamines of varying chain lengths were synthesized to investigate the possible influence of chain length and lipophilicity on antimicrobial and antibiotic-enhancing activities. The Boc-protected polyamine scaffolds **12a**–**f** (Figure 3) were synthesized using previously reported methodology [23,24,25,26].

**Scheme 2:**
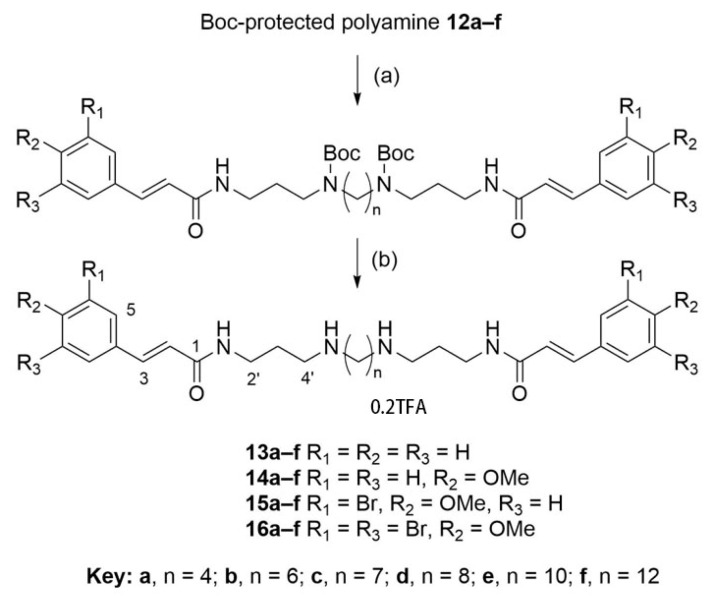
General method for the synthesis of target polyamine analogues **13**–**16**. Reagents and conditions: (a) Cinnamic acid (**5**) (2.2 equiv.), Boc-protected polyamine (**12a**–**f**) (1 equiv.), EDC·HCl/HOBt (2.6 equiv.), DIPEA (6 equiv.), CH_2_Cl_2_, rt, 20 h (yields 38–93%) or cinnamic acid (**6–8**) (2.2 equiv.), Boc-protected polyamine (**12a**–**f**) (1 equiv.), EDC·HCl (2.8 equiv.), DMAP (5 equiv.) and CH_2_Cl_2_, rt, 20 h (yields 26–88%); (b) TFA (0.2 mL) and CH_2_Cl_2_ (2 mL), N_2_, rt, 2 h (yields 27–92%).

Reaction of cinnamic acids **5**–**8** with Boc-protected polyamines **12a**–**f** utilized coupling reagents EDC·HCl or EDC·HCl/HOBt in anhydrous CH_2_Cl_2_ with the products then deprotected with TFA to afford the target compounds as their di-TFA salts (Scheme 2).

The intrinsic antimicrobial activity of the series was evaluated against a range of Gram-positive (*S. aureus* and MRSA) and Gram-negative (*E. coli*, *P. aeruginosa*, *K. pneumoniae* and *A. baumannii*) bacteria and two fungal strains (*C. albicans* and *C. neoformans*) (Table 1). Overall, the set of compounds tended to exhibit antimicrobial activity towards the Gram-positive bacteria *S. aureus* and MRSA and the fungus *C. neoformans*, and in some cases *C. albicans*, but they were only poorly active towards the Gram-negative bacteria *E. coli* and essentially inactive towards *P. aeruginosa*, *A. baumannii* and *K. pneumoniae*. More pronounced activities were observed for bromine-containing analogues **15a**–**f** and **16a**–**f** which are seemingly independent of polyamine chain length; while for those non-brominated analogues (**13a**–**f** and **14a**–**f**), the trend was that the longer the polyamine chain, the more likely was the observation of activity, e.g., **13f** and **14d**–**f**.

Cytotoxicity towards HEK293 (human kidney epithelial cell line, IC_50_) and hemolytic activity against human red blood cells (HC_10_) of the compound set was also determined (Table 1). The results trended in a similar manner to intrinsic antimicrobial activities shown in Table 1, with the longer polyamine chain analogues and those containing bromine exhibiting cytotoxicity and/or hemolysis. Interest in the promising antimicrobial activities of the cinnamido-PA-3-12-3 analogue **13f** and 4-methoxycinnamido analogues **14e** and **14f** was tempered somewhat by the observation of cytotoxicity (HEK293 IC_50_ 19.9 µM for **13f**, IC_50_ 2.3 µM for **14e** and IC_50_ 24.3 µM for **14f**) for the three analogues. The midchain-length polyamine analogue **14d** is one of the few antimicrobial analogues that does not exhibit cytotoxicity/hemolysis, illustrating in this series of compounds the fine line between a compound being antimicrobial and yet not exhibiting any mammalian cell toxicity.

We then evaluated the set of analogues for the ability to enhance the antibiotic activity of doxycycline against *P. aeruginosa* ATCC 27853 and of erythromycin against *E. coli* ATCC 25922 (Table 2). In the case of doxycycline, a fixed concentration of the antibiotic of 4.5 μM, which is twenty-fold lower than the intrinsic MIC 90 μM against this organism, was used. Each of the test compounds was then evaluated at a range of concentrations varying from 3.125 to 50–100 μM, with the upper concentration dependent upon the compounds intrinsic MIC towards *P. aeruginosa* (Table 1). The natural product ianthelliformisamine C **16a** exhibited strong 32-fold enhancement at a concentration of 5.86 µM, in close agreement with that previously reported [21]. The methylene homologues **16b** and **16c** also exhibited strong activity, at concentrations of about 11 µM. It was interesting to note that while these dibrominated analogues were active as doxycycline enhancers, no activity was observed for the mono-brominated analogues **15a**–**f**, while activity was observed for the non-brominated analogues **13b**, **13d**, **13f** and **14f**, all of which exhibited activity with comparable potency to ianthelliformisamine C **16a** and with good levels of enhancement (>64-fold, >32-fold, 8-fold and 32-fold, respectively). While the observation of cytotoxicity/hemolytic properties of **16a**–**c**, **14f** and **13f** reduces our interest in these analogues, analogues **13b** and **13d** were identified as non-toxic and strong enhancers of the action of doxycycline towards *P. aeruginosa*.

Evaluation of the compounds **13**–**16** to enhance the action of erythromycin, at a fixed dose of 8 µg/mL (10.69 μM), against *E. coli* ATCC 25922 revealed the set to be overall weakly active with the best enhancement observed for **13e** (MIC 64.5 µM, 10-fold enhancement), an analogue that was also cytotoxic and hemolytic.

**Figure 4:**
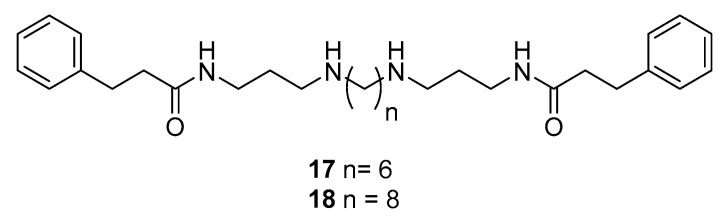
Structures of 3-phenylpropanamido-polyamines **17** and **18**.

We recently reported the results of a study exploring the antimicrobial and antibiotic potentiating activities of a series of α,ω-diacyl substituted polyamines [28]. Two of the compounds included in that study were **17** and **18** (Figure 4), which are tetrahydro analogues of **13b** and **13d**, respectively. Unlike **13b** and **13d**, compounds **17** and **18** were devoid of antimicrobial activity and the ability to enhance the action of doxycycline towards *P. aeruginosa*, highlighting the requirement of the cinnamate olefin for biological activity.

The antibiotic-enhancing activities of **13b**, the strongest non-toxic doxycycline enhancer, were further evaluated with an expanded set of antibiotics (minocycline, chloramphenicol, vancomycin, nalidixic acid and erythromycin) against three drug-resistant strains of Gram-negative bacteria (*P. aeruginosa*, *K. pneumoniae* and *A. baumannii*) (Table 3). No significant potentiating activity was observed with **13b** for chloramphenicol, vancomycin, nalidixic acid and erythromycin against the three bacterial strains. However, **13b** was able to strongly potentiate the activity of minocycline against *P. aeruginosa*, *K. pneumoniae* and *A. baumannii* (>41-fold, >82-fold and >82-fold, respectively) while also enhancing the activity of doxycycline against *P. aeruginosa* (>40-fold). The enhancing activity of doxycycline against *A. baumannii* and *K. pneumoniae* was not investigated due to its sensitivity to these strains.

## 4. Conclusions

Polyamines that are α,ω-disubstituted can exhibit antimicrobial properties and enhance the activity of legacy antibiotics towards drug-resistant Gram-negative bacteria. The present study explored structural requirements for both properties of the marine natural product ianthelliformisamine C (**4**). The majority of the analogues that contained longer chain polyamines, PA-3-8-3, PA-3-10-3 and PA-3-12-3, and any analogues containing one or two bromine atoms per capping end group were found to exhibit antimicrobial activities towards Gram-positive bacteria and the fungus *C. neoformans*. Unfortunately, these intrinsic antimicrobial activities were usually associated with cytotoxicity and/or red blood cell hemolytic properties. Of more promise was the observation of 32-fold or better levels of enhancement of the action of doxycycline towards the Gram-negative bacterial pathogen *P. aeruginosa* for simple cinnamido-polyamine analogues **13b** and **13d**, with the former also enhancing the activity of minocycline against *K. pneumoniae* and *A. baumannii*. Comparison with previously reported compounds determined the essentiality of the olefin group for activity, thus any future studies to optimize this class of antibiotic enhancers must retain the core cinnamide structure but avoid incorporation of halogen and methoxyl groups at the meta and para-aryl positions.

## Figures and Tables

**Table 1:** Antimicrobial activities (MIC, µM), cytotoxicity (IC_50_, µM) and hemolytic activity (HC_10_, µM) of analogues **13**–**16**.

Compound	MIC (µM)						Cytotoxicity ^g^	Hemolysis ^h^
	*S. a* ^a^	MRSA ^b^	*P. a* ^c^	*E. c* ^d^	*C. a* ^e^	*C. n* ^f^		
**13a**	724	>46.3	724	724	>46.3	>46.3	n.t. ^i^	n.t. ^i^
**13b**	>200	>44.5	>200	100	>44.5	44.5	>44.5	>44.5
**13c**	>273	>43.7	>273	>273	>43.7	>43.7	>43.7	>43.7
**13d**	100	42.8	>200	100	>42.8	42.8	>42.9	>42.9
**13e**	64.5	20.6	645	645	>41.3	41.3	24.3	37.6
**13f**	3.125	≤0.31	50	1.56	>39.9	≤0.31	19.9	>39.9
**14a**	33.3	>42.6	>266	266	>42.6	n.t.^i^	>42.6	>42.6
**14b**	64.2	41.1	>257	>257	>41.1	≤0.32	>41.1	>41.1
**14c**	31.5	20.2	>252	252	>40.4	n.t. ^i^	>40.4	>40.4
**14d**	7.75	≤0.31	248	62	>39.7	n.t. ^i^	>39.7	>39.7
**14e**	1.87	≤0.30	240	120	38.3	≤0.30	2.3	>38.3
**14f**	3.62	≤0.29	232	7.24	>37.1	n.t. ^i^	24.3	n.t. ^i^
**15a**	13.8	2.2	>220	55	17.6	≤0.28	>35.2	n.t. ^i^
**15b**	6.67	2.1	>214	53	>34.1	≤0.27	>34.2	0.99
**15c**	6.57	≤0.26	>210	26	8.4	≤0.26	>33.7	n.t. ^i^
**15d**	6.48	≤0.26	>207	25.9	33.2	≤0.26	19.7	0.52
**15e**	1.57	≤0.25	50.4	25.2	32.2	1.0	>32.2	24.1
**15f**	6.12	≤0.24	>196	6.12	n.t. ^i^	≤0.24	1.12	1.17
**16a**	5.86	≤0.23	188	11.7	30	≤0.23	>30	≤0.23
**16b**	5.71	≤0.23	>183	11.4	29.2	≤0.23	15.6	0.91
**16c**	5.64	≤0.23	180	11.3	>28.9	≤0.23	22.0	0.31
**16d**	5.57	≤0.22	>178	11.1	>28.5	≤0.22	11.0	≤0.22
**16e**	1.36	≤0.22	87	43.5	27.8	≤0.22	2.0	13.6
**16f**	5.30	≤0.21	>170	5.30	≤0.21	≤0.21	1.44	0.24

^a^ *S. aureus* ATCC 25923 with streptomycin (MIC 21.5 μM) and chloramphenicol (MIC 1.5–3 μM) as positive controls and values presented as the mean (n = 3); ^b^ MRSA ATCC 43300 with vancomycin (MIC 0.7 μM) as the positive control and values presented as the mean (n = 2); ^c^ *P. aeruginosa* ATCC 27853 with streptomycin (MIC 21.5 μM) and colistin (MIC 1 μM) as positive controls and values presented as the mean (n = 3); ^d^ *E. coli* ATCC 25922 with streptomycin (MIC 21.5 μM) and colistin (MIC 2 μM) as positive controls and values presented as the mean (n = 3); ^e^ *C. albicans* ATCC 90028 with fluconazole (MIC 0.4 μM) as the positive control and values presented as the mean (n = 2); ^f^ *C. neoformans* ATCC 208821 with fluconazole (MIC 26 μM) as the positive control and values presented as the mean (n = 2); ^g^ Concentration of compound at 50% cytotoxicity on HEK293 human embryonic kidney cells with tamoxifen as the positive control (IC_50_ 24 μM) and values presented as the mean (n = 2); ^h^ Concentration of compound at 10% hemolytic activity on human red blood cells with melittin as the positive control (HC_10_ 0.95 μM) and values presented as the mean (n = 2); ^i^ Not tested.

**Table 2:** Antibiotic enhancement activity (MIC, µM) of analogues **13**–**16**.

Compound	Dox/*P. a* ^a^	Eryth/*E. c* ^b^
**13a**	145 (5)	145 (5)
**13b**	3.125 (>64)	25 (4)
**13c**	68 (>4)	136 (>2)
**13d**	6.25 (>32)	25 (4)
**13e**	645 (1)	64.5 (10)
**13f**	6.25 (8)	3.125 (0.5)
**14a**	16.6 (>16)	67 (4)
**14b**	64 (>4)	256 (>1)
**14c**	31.5 (>8)	126 (2)
**14d**	15.5 (16)	62 (1)
**14e**	60 (4)	n.t. ^c^
**14f**	7.24 (32)	3.62 (2)
**15a**	27.5 (>8)	13.7 (4)
**15b**	53.4 (>4)	26.7 (2)
**15c**	26.3 (>8)	13.1 (2)
**15d**	51.8 (>4)	25.9 (1)
**15e**	50.4 (1)	n.t. ^c^
**15f**	49.0 (>4)	6.1 (1)
**16a**	5.86 (32)	11.7 (1)
**16b**	11.4 (>16)	11.4 (1)
**16c**	11.3 (16)	22.6 (0.5)
**16d**	44.5 (>4)	22.3 (0.5)
**16e**	43.5 (2)	n.t. ^c^
**16f**	42.4 (>4)	5.30 (1)

^a^ Concentration (µM) required to restore doxycycline activity at 4.5 µM against *P. aeruginosa* ATCC 27853. Fold change shown in parentheses is the ratio between the intrinsic MIC of the test compound and the combination MIC; ^b^ Concentration (µM) required to restore erythromycin activity at 10.9 µM against *E. coli* ATCC 25922. Fold change shown in parentheses is the ratio between the intrinsic MIC of the test compound and the combination MIC; ^c^ Not tested.

**Table 3:** Antibiotic potentiating activity of **13b**.

Antibiotic	Concentration (µM) for Potentiation ^a^		
	*P. a* ^b^	*K. p* ^c^	*A. b* ^d^
No antibiotic	>278	>278	>278
Doxycycline	7.03 (>40)	n.d. ^e^	n.d. ^e^
Minocycline	6.83 (>41)	3.41 (>82)	3.41 (>82)
Chloramphenicol	38.7 (>7)	>200	77.4 (>4)
Vancomycin	n.d. ^e^	138 (>2)	138 (>2)
Nalidixic acid	n.d. ^e^	>200	>200
Erythromycin	136 (>2)	>200	136 (>2)

^a^ Concentration (µM) of compound required to restore antibiotics activity at 2 µg/mL concentration of antibiotic and values presented as the mean (n = 2). Fold change shown in parentheses is the ratio between the intrinsic MIC of the test compound and the combination MIC; ^b^ *P. aeruginosa* PAO1 against doxycycline (MIC 144 μM), minocycline (MIC 70 μM), chloramphenicol (MIC > 200 µM), vancomycin (MIC 138 μM), nalidixic acid (MIC > 200 μM) and erythromycin (MIC > 200 µM); ^c^ *K. pneumoniae* ATCC 13883 against minocycline (MIC 27 μM), chloramphenicol (MIC > 200 µM), vancomycin (MIC 138 µM), nalidixic acid (MIC > 200 μM) and erythromycin (MIC > 200 µM); ^d^ *A. baumannii* AYE against minocycline (MIC 27 μM), chloramphenicol (MIC > 200 µM), vancomycin (MIC 138 µM), nalidixic acid (MIC > 200 μM) and erythromycin (MIC > 200 µM); ^e^ Not determined.

## Data Availability

Data are contained within the article or Supplementary Materials.

## Supplementary Materials

The following supporting information can be downloaded at: https://www.mdpi.com/article/10.3390/biom13071087/s1, Figure S1: ^1^H (CD_3_OD, 400 MHz) and ^13^C (CD_3_OD, 100 MHz) NMR spectra for **13a**; Figure S2: ^1^H (CD_3_OD, 400 MHz) and ^13^C (CD_3_OD, 100 MHz) NMR spectra for **13b**; Figure S3: ^1^H (CD_3_OD, 400 MHz) and ^13^C (CD_3_OD, 100 MHz) NMR spectra for **13c**; Figure S4: ^1^H (CD_3_OD, 400 MHz) and ^13^C (CD_3_OD, 100 MHz) NMR spectra for **13d**; Figure S5: ^1^H (CD_3_OD, 400 MHz) and ^13^C (CD_3_OD, 100 MHz) NMR spectra for **13e**; Figure S6: ^1^H (CD_3_OD, 400 MHz) and ^13^C (CD_3_OD, 100 MHz) NMR spectra for **13f**; Figure S7: ^1^H (DMSO-*d*_6_, 400 MHz) and ^13^C (DMSO-*d*_6_, 100 MHz) NMR spectra for **14a**; Figure S8: ^1^H (DMSO-*d*_6_, 400 MHz) and ^13^C (DMSO-*d*_6_, 100 MHz) NMR spectra for **14b**; Figure S9: ^1^H (DMSO-*d*_6_, 400 MHz) and ^13^C (DMSO-*d*_6_, 100 MHz) NMR spectra for **14c**; Figure S10: ^1^H (DMSO-*d*_6_, 400 MHz) and ^13^C (DMSO-*d*_6_, 100 MHz) NMR spectra for **14d**; Figure S11: ^1^H (DMSO-*d*_6_, 400 MHz) and ^13^C (DMSO-*d*_6_, 100 MHz) NMR spectra for **14e**; Figure S12: ^1^H (DMSO-*d*_6_, 400 MHz) and ^13^C (DMSO-*d*_6_, 100 MHz) NMR spectra for **14f**; Figure S13: ^1^H (DMSO-*d*_6_, 400 MHz) and ^13^C (DMSO-*d*_6_, 100 MHz) NMR spectra for **15a**; Figure S14: ^1^H (DMSO-*d*_6_, 400 MHz) and ^13^C (DMSO-*d*_6_, 100 MHz) NMR spectra for **15d**; Figure S15: ^1^H (DMSO-*d*_6_, 400 MHz) and ^13^C (DMSO-*d*_6_, 100 MHz) NMR spectra for **15c**; Figure S16: ^1^H (DMSO-*d*_6_, 400 MHz) and ^13^C (DMSO-*d*_6_, 100 MHz) NMR spectra for **15d**; Figure S17: ^1^H (DMSO-*d*_6_, 400 MHz) and ^13^C (DMSO-*d*_6_, 100 MHz) NMR spectra for **15e**; Figure S18: ^1^H (DMSO-*d*_6_, 400 MHz) and ^13^C (DMSO-*d*_6_, 100 MHz) NMR spectra for **15f**; Figure S19: ^1^H (DMSO-*d*_6_, 400 MHz) and ^13^C (DMSO-*d*_6_, 100 MHz) NMR spectra for **16a**; Figure S20: ^1^H (DMSO-*d*_6_, 400 MHz) and ^13^C (DMSO-*d*_6_, 100 MHz) NMR spectra for **16b**; Figure S21: ^1^H (DMSO-*d*_6_, 400 MHz) and ^13^C (DMSO-*d*_6_, 100 MHz) NMR spectra for **16c**; Figure S22: ^1^H (DMSO-*d*_6_, 400 MHz) and ^13^C (DMSO-*d*_6_, 100 MHz) NMR spectra for **16d**; Figure S23: ^1^H (DMSO-*d*_6_, 400 MHz) and ^13^C (DMSO-*d*_6_, 100 MHz) NMR spectra for **16e**; Figure S24: ^1^H (DMSO-*d*_6_, 400 MHz) and ^13^C (DMSO-*d*_6_, 100 MHz) NMR spectra for **16f**.
