# Supplementary material for: Exploration of Bis-Cinnamido-Polyamines as Intrinsic Antimicrobial Agents and Antibiotic Enhancers
Source: Biomolecules. 2023 Jul 7;13(7):1087. doi: 10.3390/biom13071087 (PMC10377643; doi:10.3390/biom13071087)
Supplement: Supplementary file 1 [file biomolecules-13-01087-s001.zip › biomolecules-2450684-supplementary.pdf]

Supporting Information

Exploration of Bis-Cinnamido-Polyamines as Intrinsic Antimicrobial Agents and Antibiotic Enhancers

Melissa M. Cadelis [1,2], Jisoo Kim [2], Florent Rouvier [3], Evangelene S. Gill [2], Kyle Fraser [2], Marie-Lise Bourguet-Kondracki [4], Jean Michel Brunel [3] and Brent R. Copp [2,*]

1 Department of Molecular Medicine and Pathology, School of Medical Sciences, The University of Auckland, Private Bag 92019, Auckland 1142, New Zealand
2 School of Chemical Sciences, The University of Auckland, Private Bag 92019, Auckland 1142, New Zealand
3 Membranes et Cibles Therapeutiques (MCT), SSA, INSERM, Aix-Marseille Universite, 27 bd Jean Moulin, 13385 Marseille, France
4 Laboratoire Molécules de Communication et Adaptation des Micro-Organismes, UMR 7245 CNRS, Muséum National d'Histoire Naturelle, 57 Rue Cuvier (C.P. 54), 75005 Paris, France
* Correspondence: b.copp@auckland.ac.nz

Contents

Figure S1 ^{1}H (CD_3OD, 400 MHz) and ^{13}C (CD_3OD, 100 MHz) NMR spectra for **13a** S3
Figure S2 ^{1}H (CD_3OD, 400 MHz) and ^{13}C (CD_3OD, 100 MHz) NMR spectra for **13b** S4
Figure S3 ^{1}H (CD_3OD, 400 MHz) and ^{13}C (CD_3OD, 100 MHz) NMR spectra for **13c** S5
Figure S4 ^{1}H (CD_3OD, 400 MHz) and ^{13}C (CD_3OD, 100 MHz) NMR spectra for **13d** S6
Figure S5 ^{1}H (CD_3OD, 400 MHz) and ^{13}C (CD_3OD, 100 MHz) NMR spectra for **13e** S7
Figure S6 ^{1}H (CD_3OD, 400 MHz) and ^{13}C (CD_3OD, 100 MHz) NMR spectra for **13f** S8
Figure S7 ^{1}H (DMSO-d_6, 400 MHz) and ^{13}C (DMSO-d_6, 100 MHz) NMR spectra for **14a** S9
Figure S8 ^{1}H (DMSO-d_6, 400 MHz) and ^{13}C (DMSO-d_6, 100 MHz) NMR spectra for **14b** S10
Figure S9 ^{1}H (DMSO-d_6, 400 MHz) and ^{13}C (DMSO-d_6, 100 MHz) NMR spectra for **14c** S11
Figure S10 ^{1}H (DMSO-d_6, 400 MHz) and ^{13}C (DMSO-d_6, 100 MHz) NMR spectra for **14d** S12
Figure S11 ^{1}H (DMSO-d_6, 400 MHz) and ^{13}C (DMSO-d_6, 100 MHz) NMR spectra for **14e** S13
Figure S12 ^{1}H (DMSO-d_6, 400 MHz) and ^{13}C (DMSO-d_6, 100 MHz) NMR spectra for **14f** S14
Figure S13 ^{1}H (DMSO-d_6, 400 MHz) and ^{13}C (DMSO-d_6, 100 MHz) NMR spectra for **15a** S15
Figure S14 ^{1}H (DMSO-d_6, 400 MHz) and ^{13}C (DMSO-d_6, 100 MHz) NMR spectra for **15b** S16
Figure S15 ^{1}H (DMSO-d_6, 400 MHz) and ^{13}C (DMSO-d_6, 100 MHz) NMR spectra for **15c** S17
Figure S16 ^{1}H (DMSO-d_6, 400 MHz) and ^{13}C (DMSO-d_6, 100 MHz) NMR spectra for **15d** S18
Figure S17 ^{1}H (DMSO-d_6, 400 MHz) and ^{13}C (DMSO-d_6, 100 MHz) NMR spectra for **15e** S19
Figure S18 ^{1}H (DMSO-d_6, 400 MHz) and ^{13}C (DMSO-d_6, 100 MHz) NMR spectra for **15f** S20
Figure S19 ^{1}H (DMSO-d_6, 400 MHz) and ^{13}C (DMSO-d_6, 100 MHz) NMR spectra for **16a** S21

Figure S20 ^{1}H (DMSO-d_6, 400 MHz) and ^{13}C (DMSO-d_6, 100 MHz) NMR spectra for **16b** **S22**
Figure S21 ^{1}H (DMSO-d_6, 400 MHz) and ^{13}C (DMSO-d_6, 100 MHz) NMR spectra for **16c** **S23**
Figure S22 ^{1}H (DMSO-d_6, 400 MHz) and ^{13}C (DMSO-d_6, 100 MHz) NMR spectra for **16d** **S24**
Figure S23 ^{1}H (DMSO-d_6, 400 MHz) and ^{13}C (DMSO-d_6, 100 MHz) NMR spectra for **16e** **S25**
Figure S24 ^{1}H (DMSO-d_6, 400 MHz) and ^{13}C (DMSO-d_6, 100 MHz) NMR spectra for **16f** **S26**

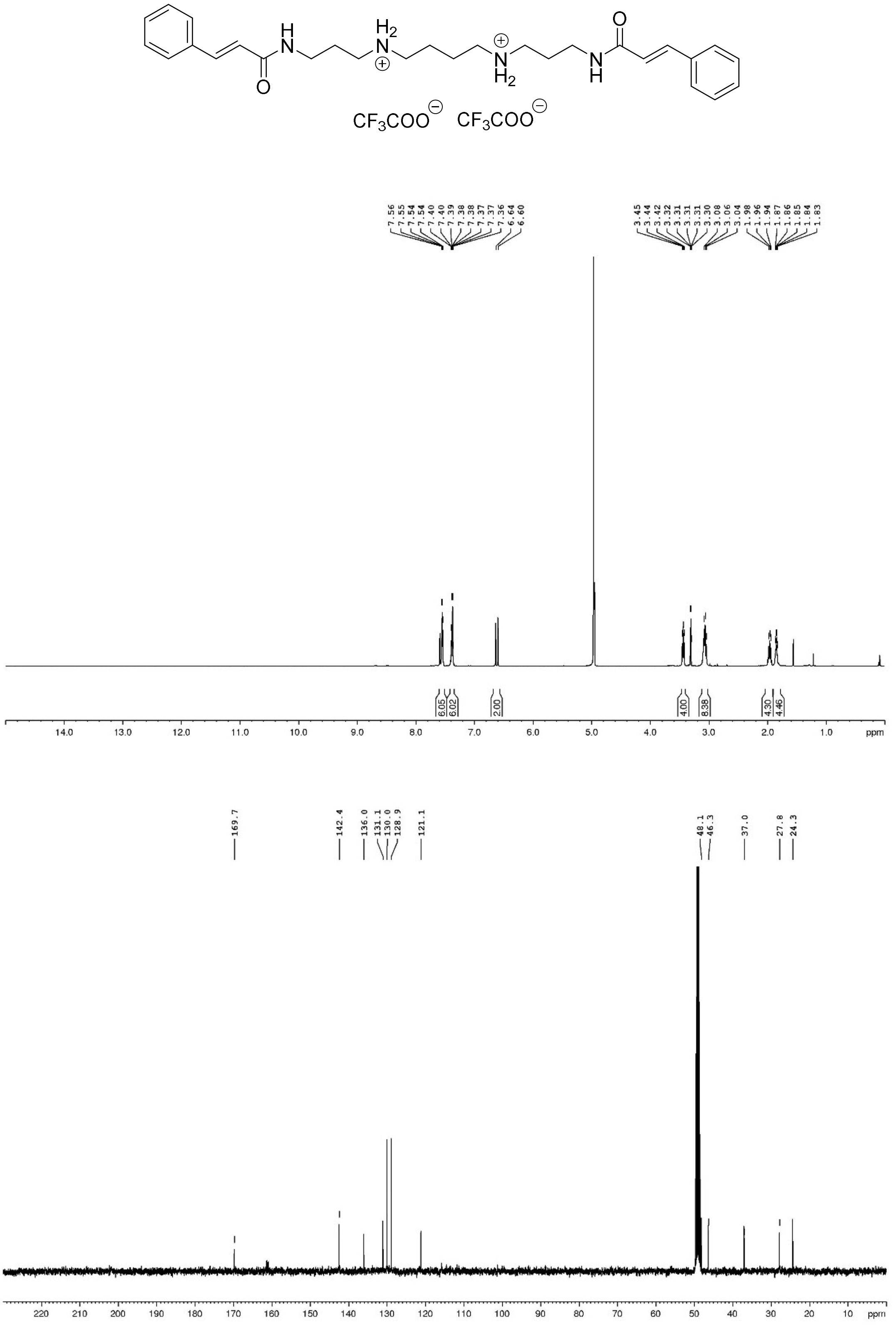

Figure S1. ^{1}H (CD_3OD, 400 MHz) and ^{13}C (CD_3OD, 100 MHz) NMR spectra for **13a**.

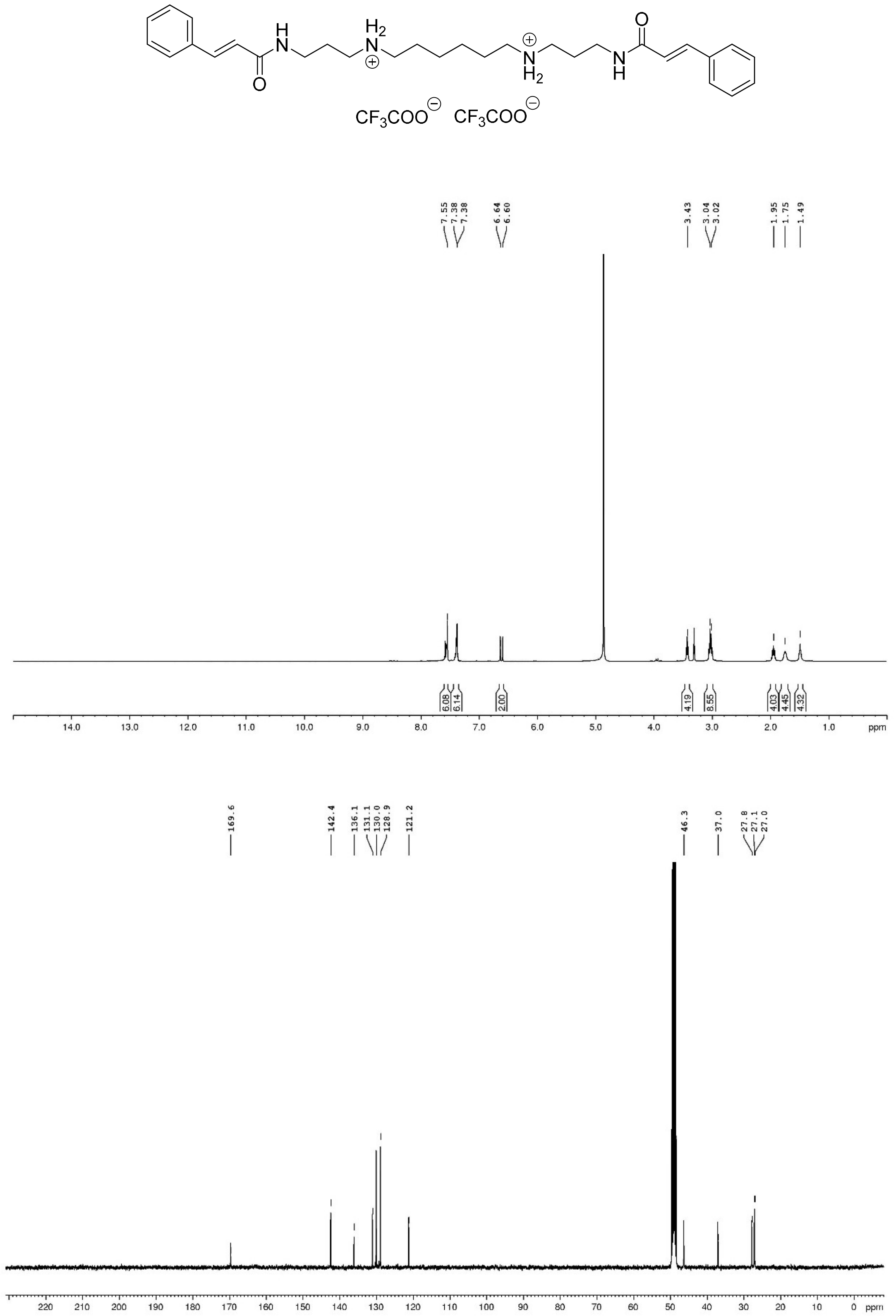

Figure S2. ^{1}H (CD_3OD, 400 MHz) and ^{13}C (CD_3OD, 100 MHz) NMR spectra for **13b**.

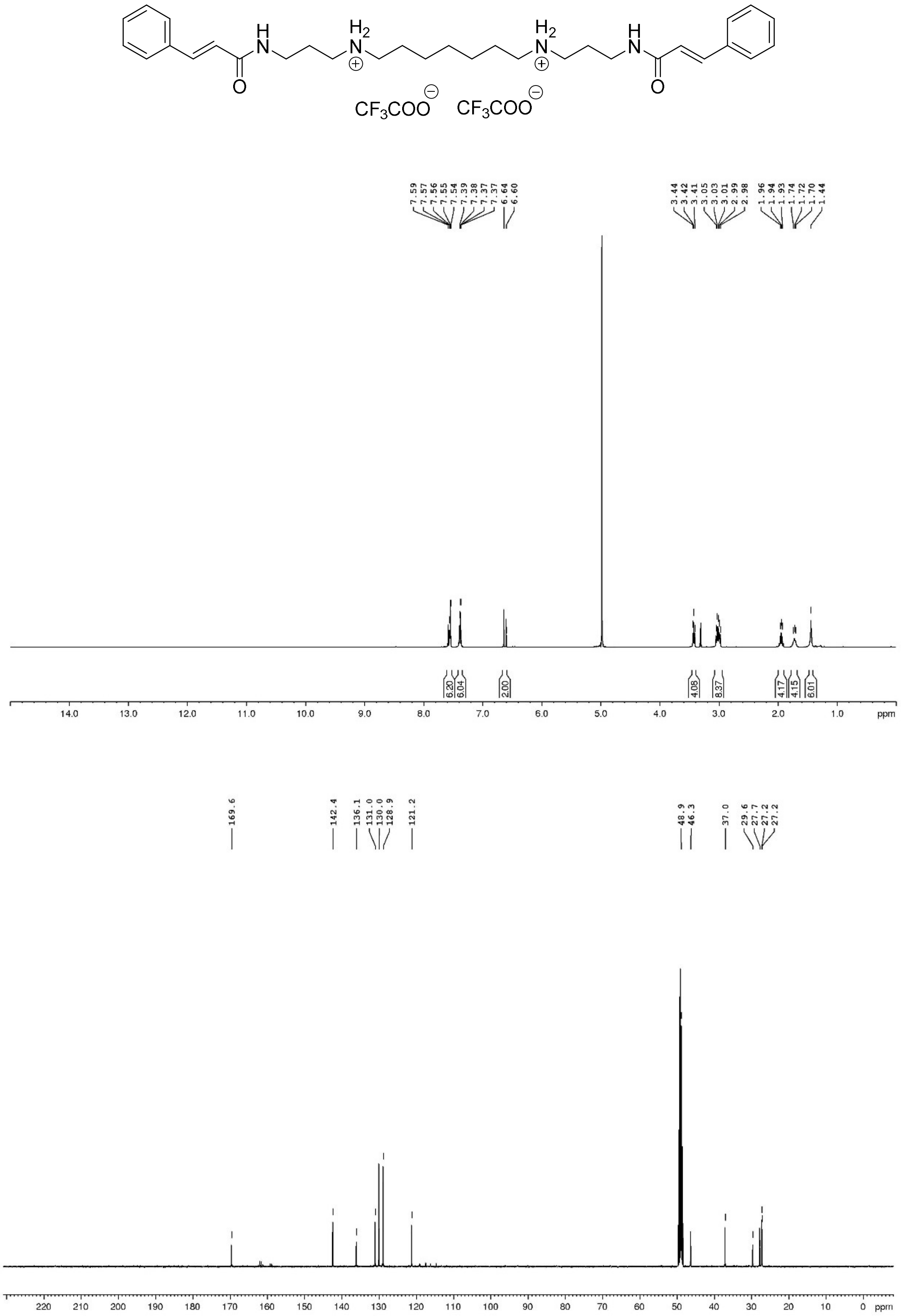

Figure S3. ^{1}H (CD_3OD, 400 MHz) and ^{13}C (CD_3OD, 100 MHz) NMR spectra for **13c**.

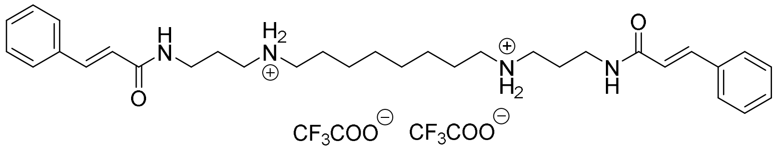

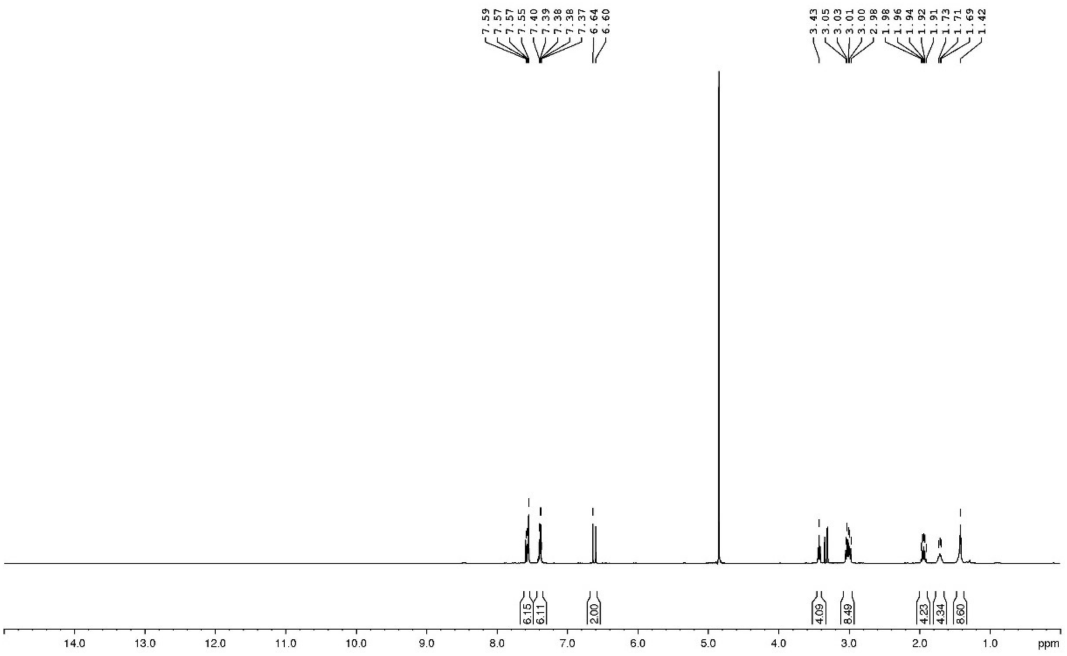

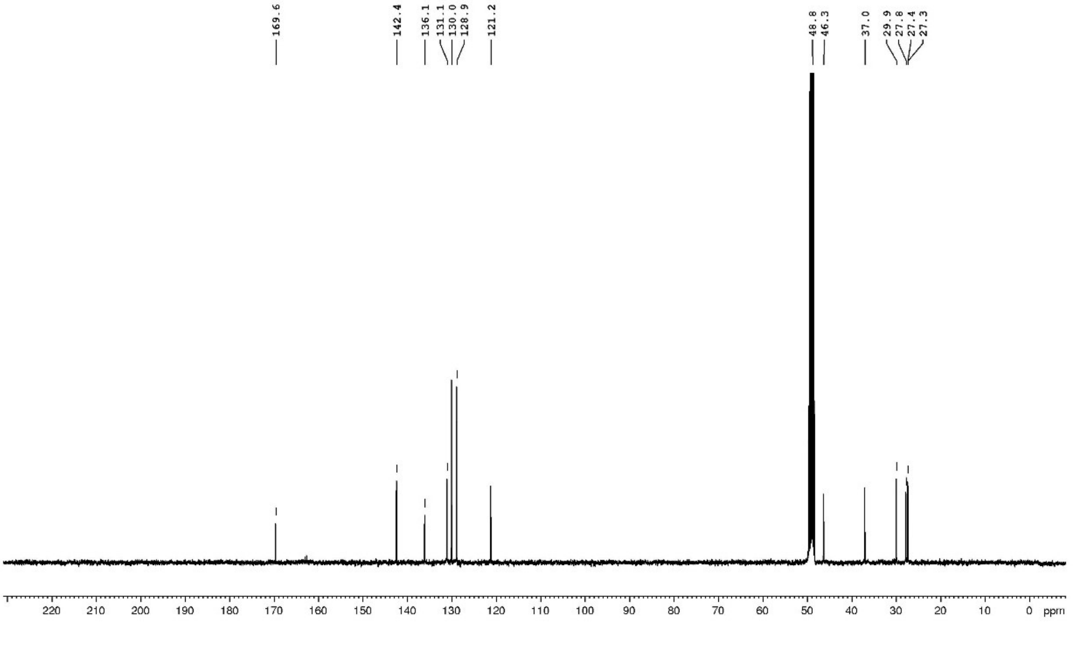

Figure S4. ^{1}H (CD_3OD, 400 MHz) and ^{13}C (CD_3OD, 100 MHz) NMR spectra for **13d**.

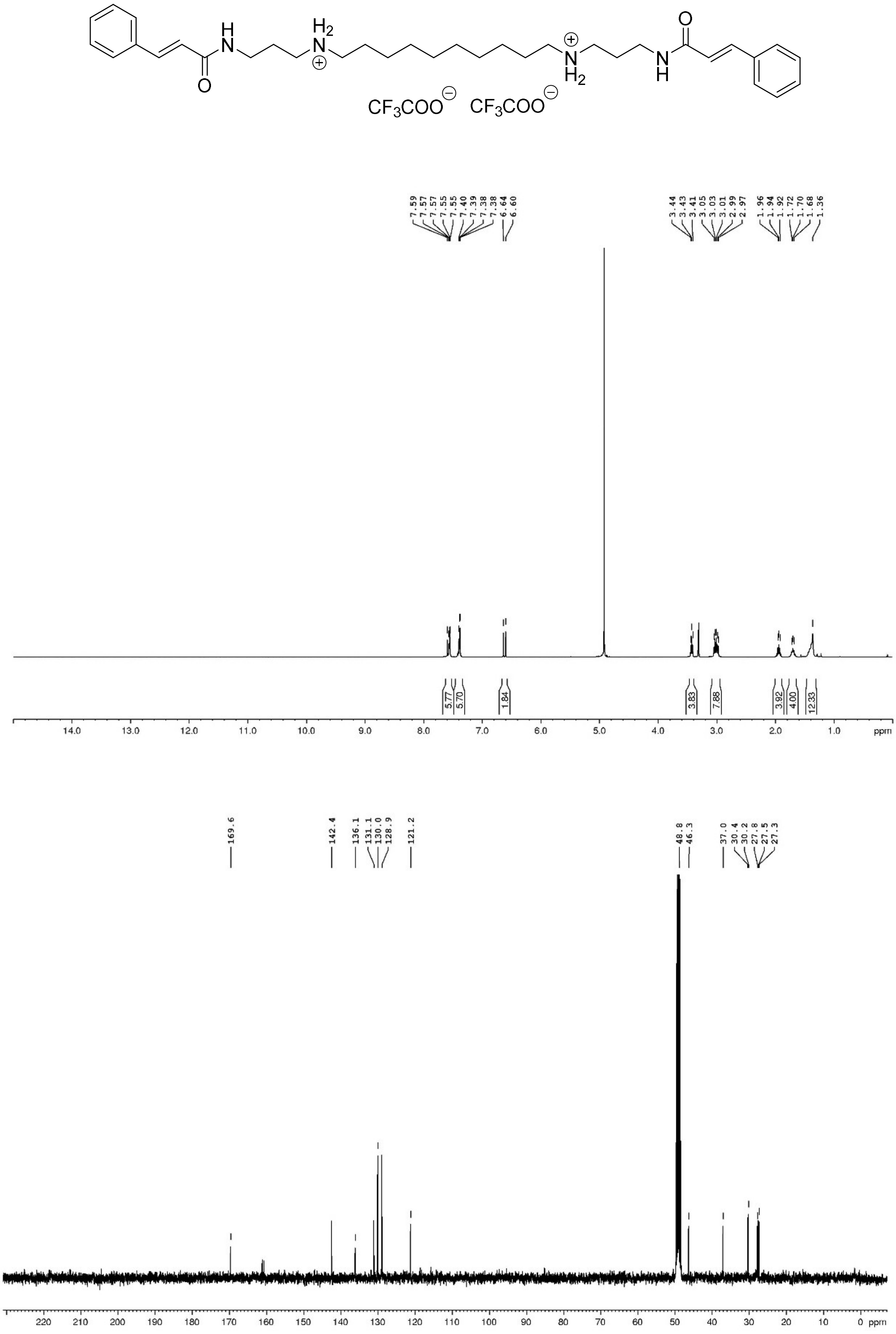

Figure S5. ^{1}H (CD_3OD, 400 MHz) and ^{13}C (CD_3OD, 100 MHz) NMR spectra for **13e**.

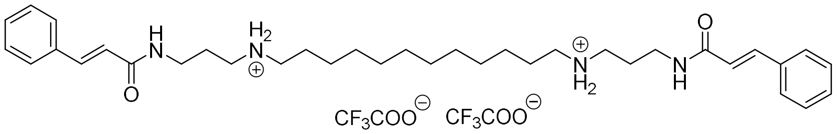

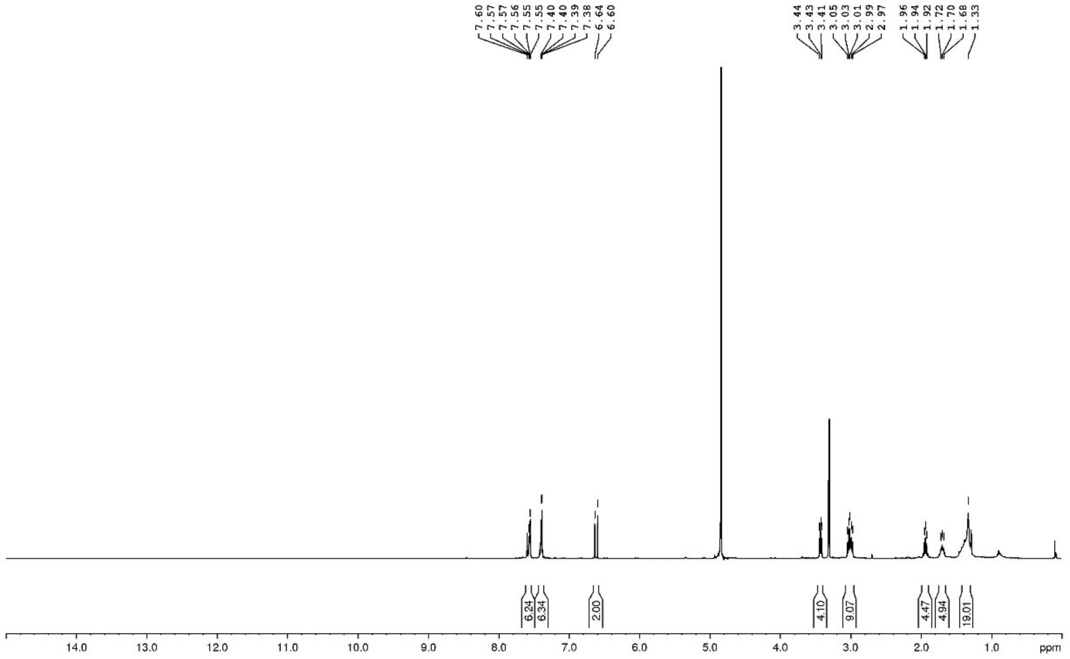

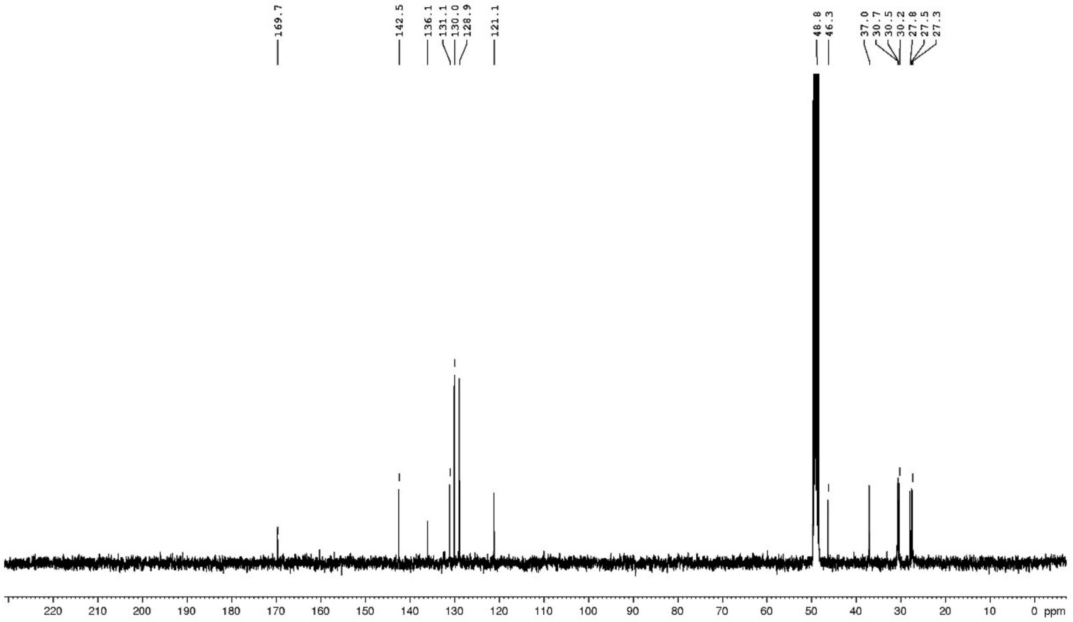

Figure S6. ^{1}H (CD_3OD, 400 MHz) and ^{13}C (CD_3OD, 100 MHz) NMR spectra for **13f**.

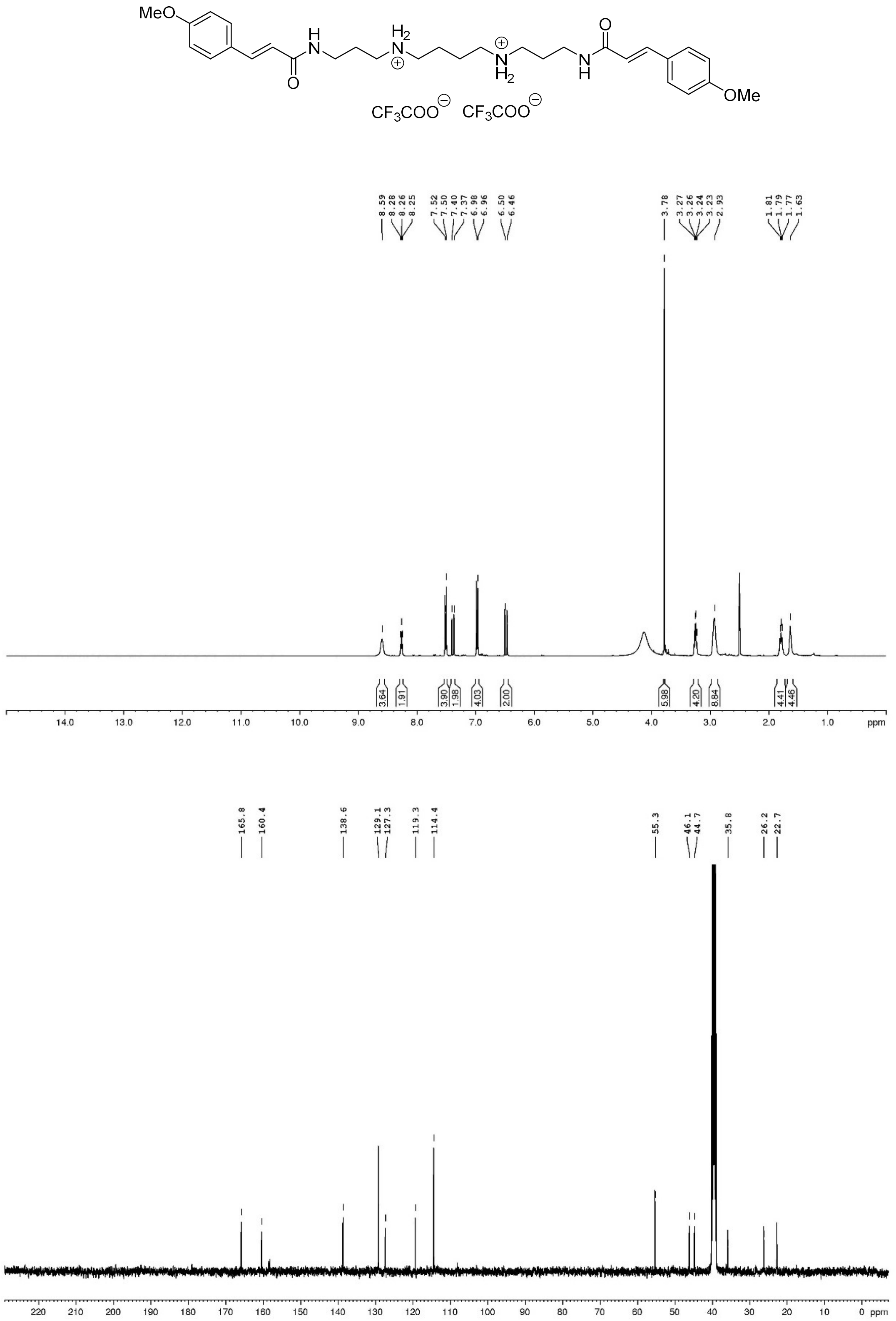

Figure S7. ^{1}H (DMSO-d_6, 400 MHz) and ^{13}C (DMSO-d_6, 100 MHz) NMR spectra for **14a**.

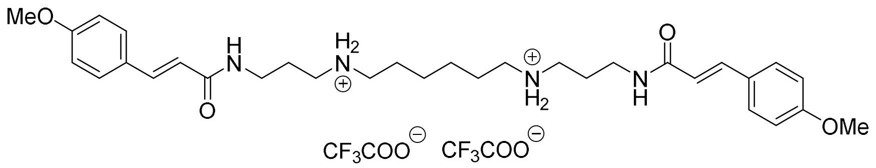

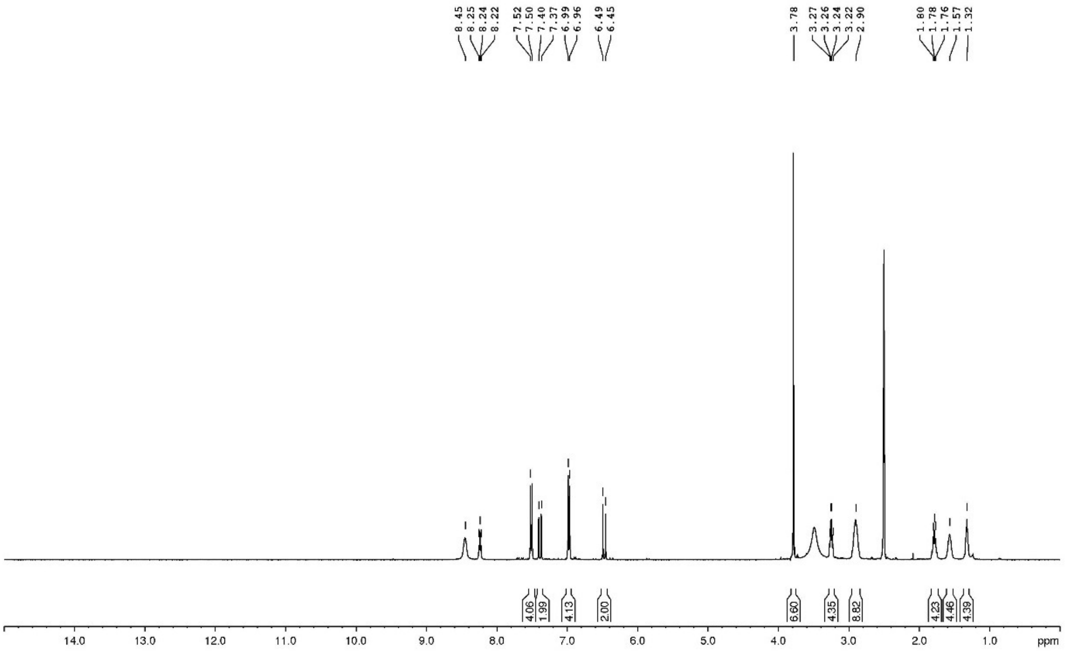

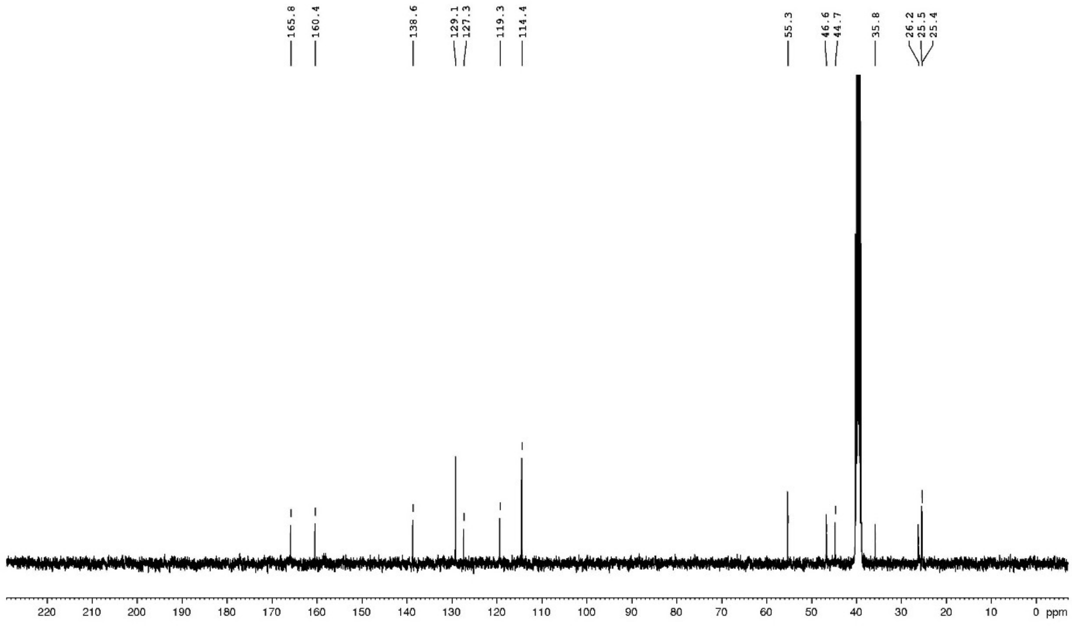

Figure S8. ^{1}H (DMSO-d_6, 400 MHz) and ^{13}C (DMSO-d_6, 100 MHz) NMR spectra for **14b**.

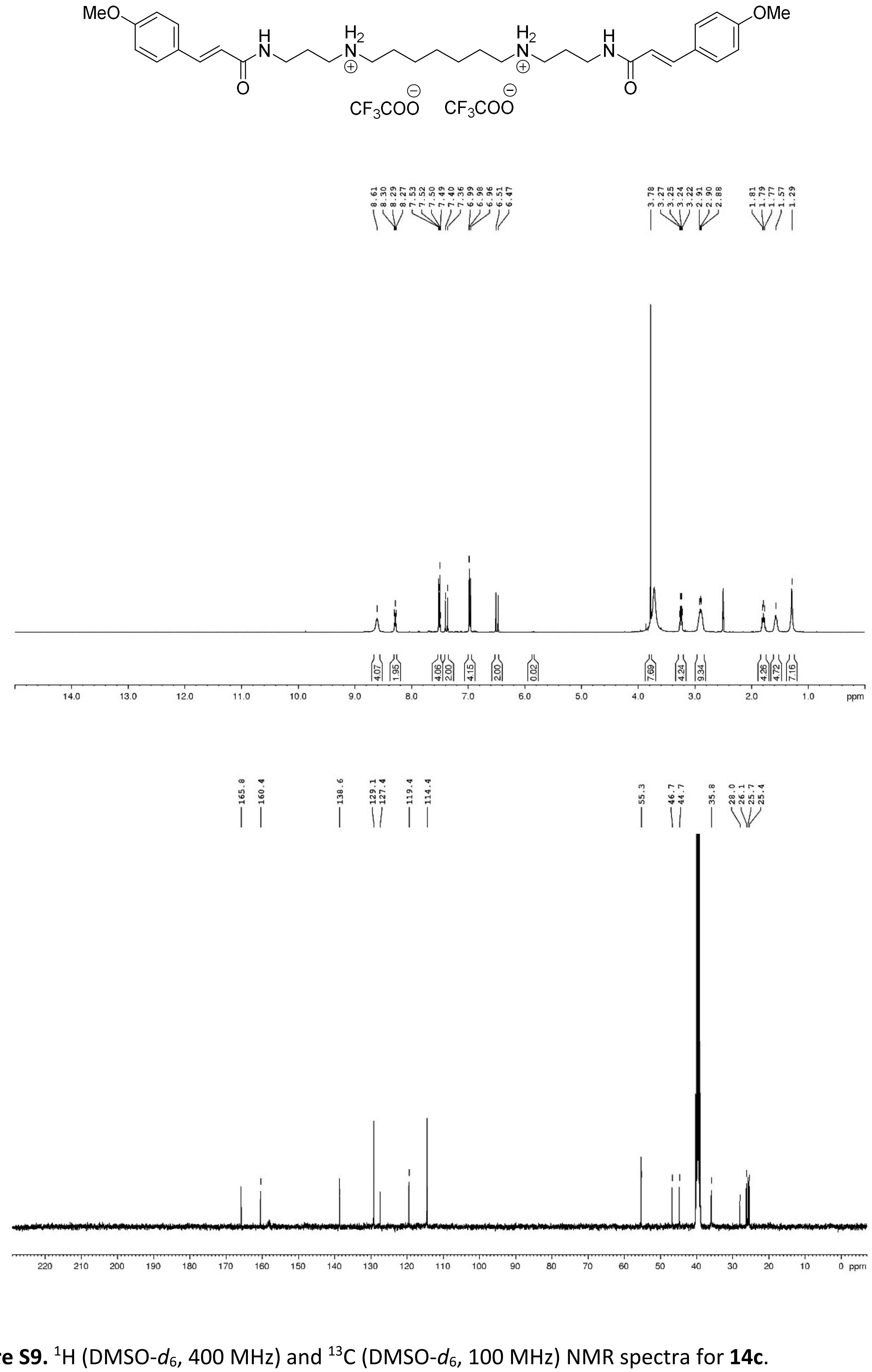

Figure S9. ^{1}H (DMSO-d_6, 400 MHz) and ^{13}C (DMSO-d_6, 100 MHz) NMR spectra for **14c**.

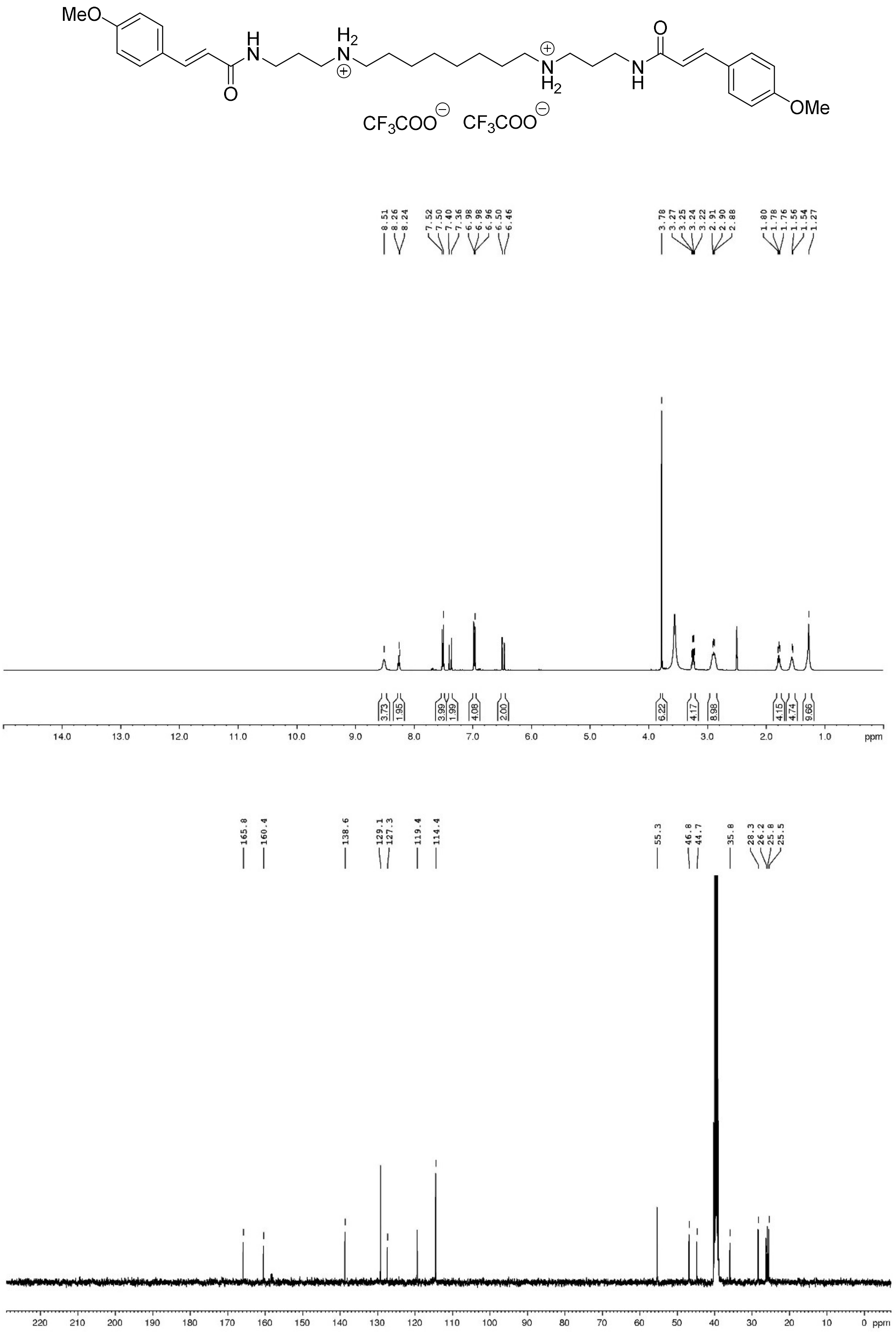

Figure S10. ^{1}H (DMSO-d_6, 400 MHz) and ^{13}C (DMSO-d_6, 100 MHz) NMR spectra for **14d**.

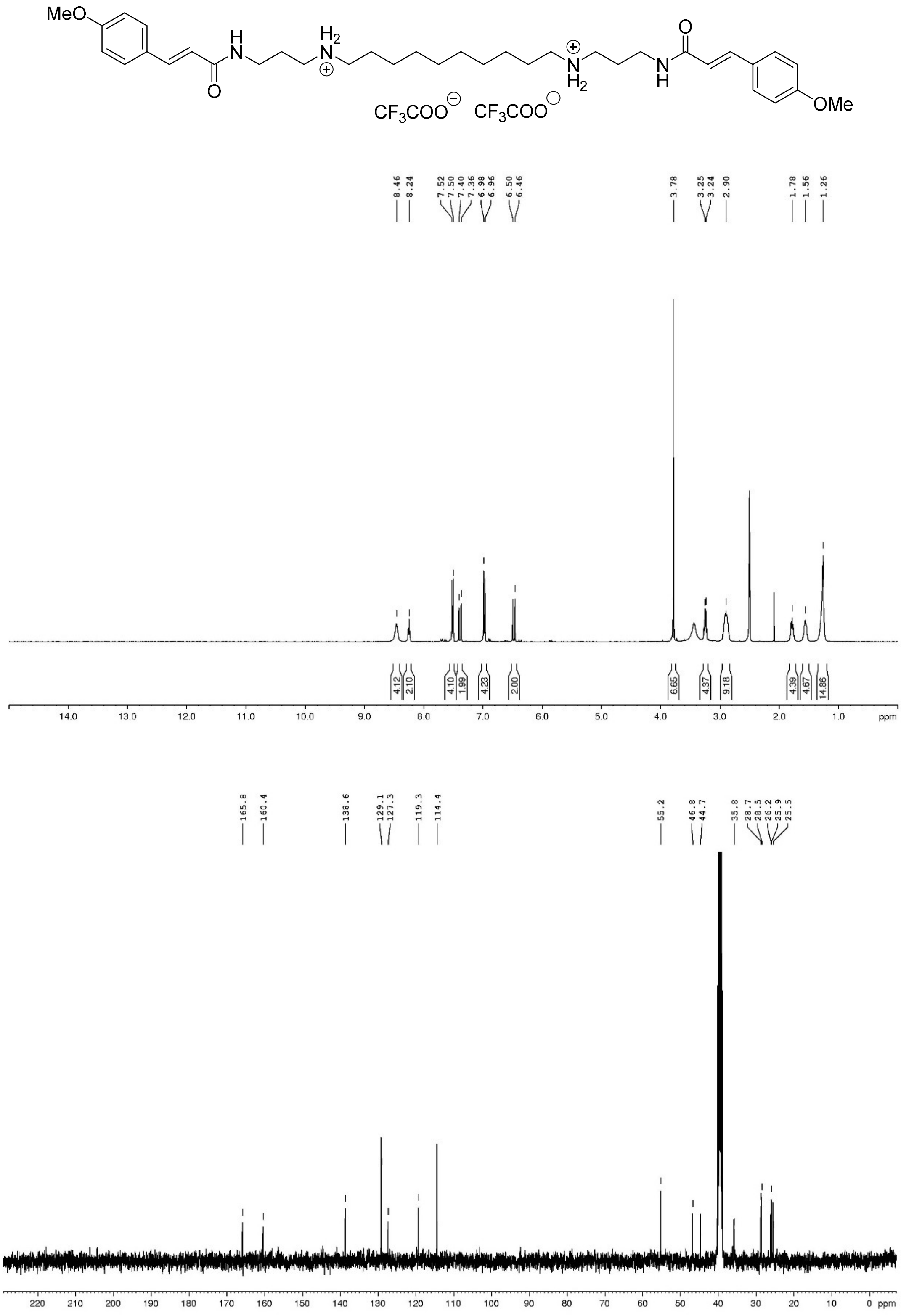

Figure S11. ^{1}H (DMSO-d_6, 400 MHz) and ^{13}C (DMSO-d_6, 100 MHz) NMR spectra for **14e**.

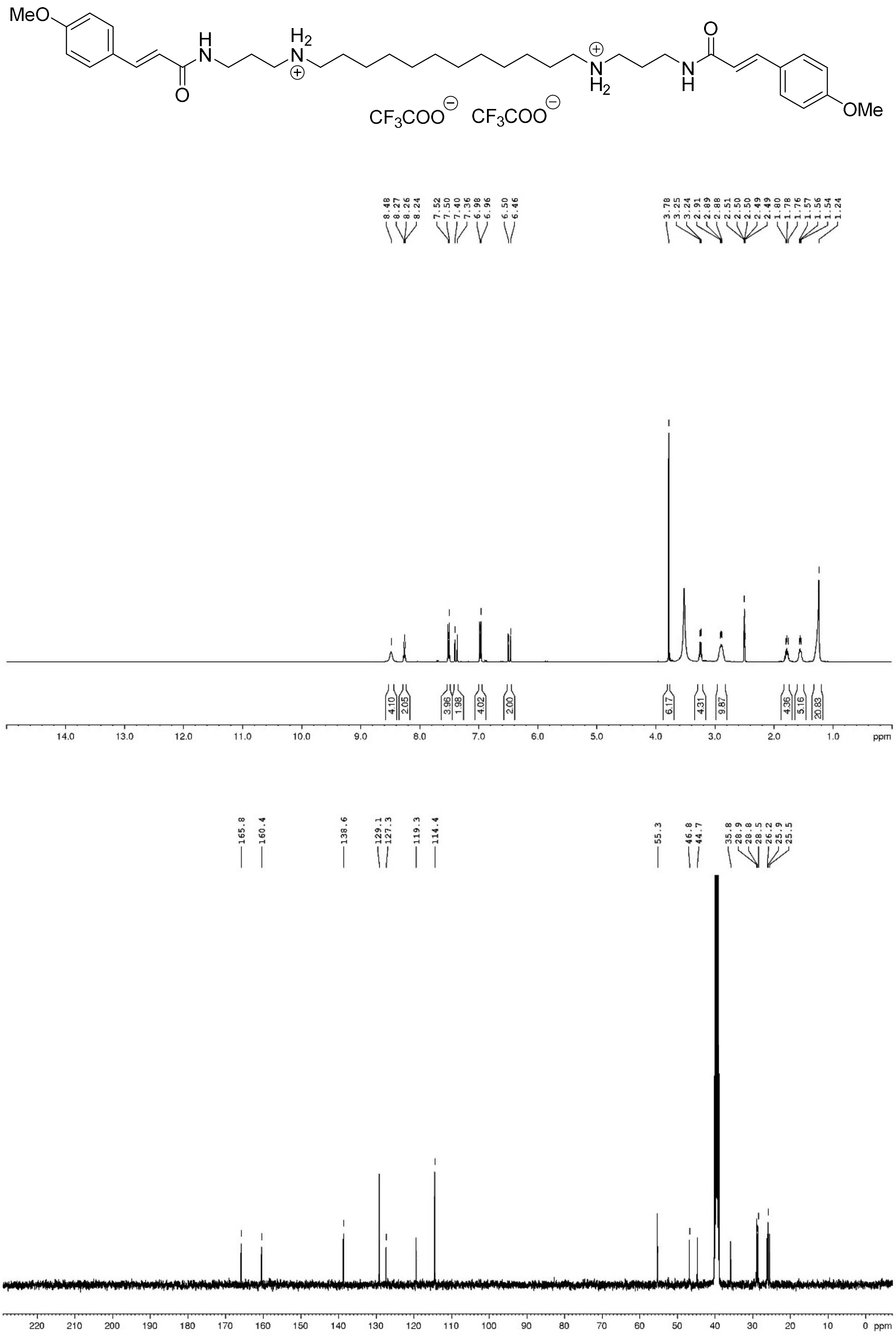

Figure S12. ^{1}H (DMSO-d_6, 400 MHz) and ^{13}C (DMSO-d_6, 100 MHz) NMR spectra for **14f**.

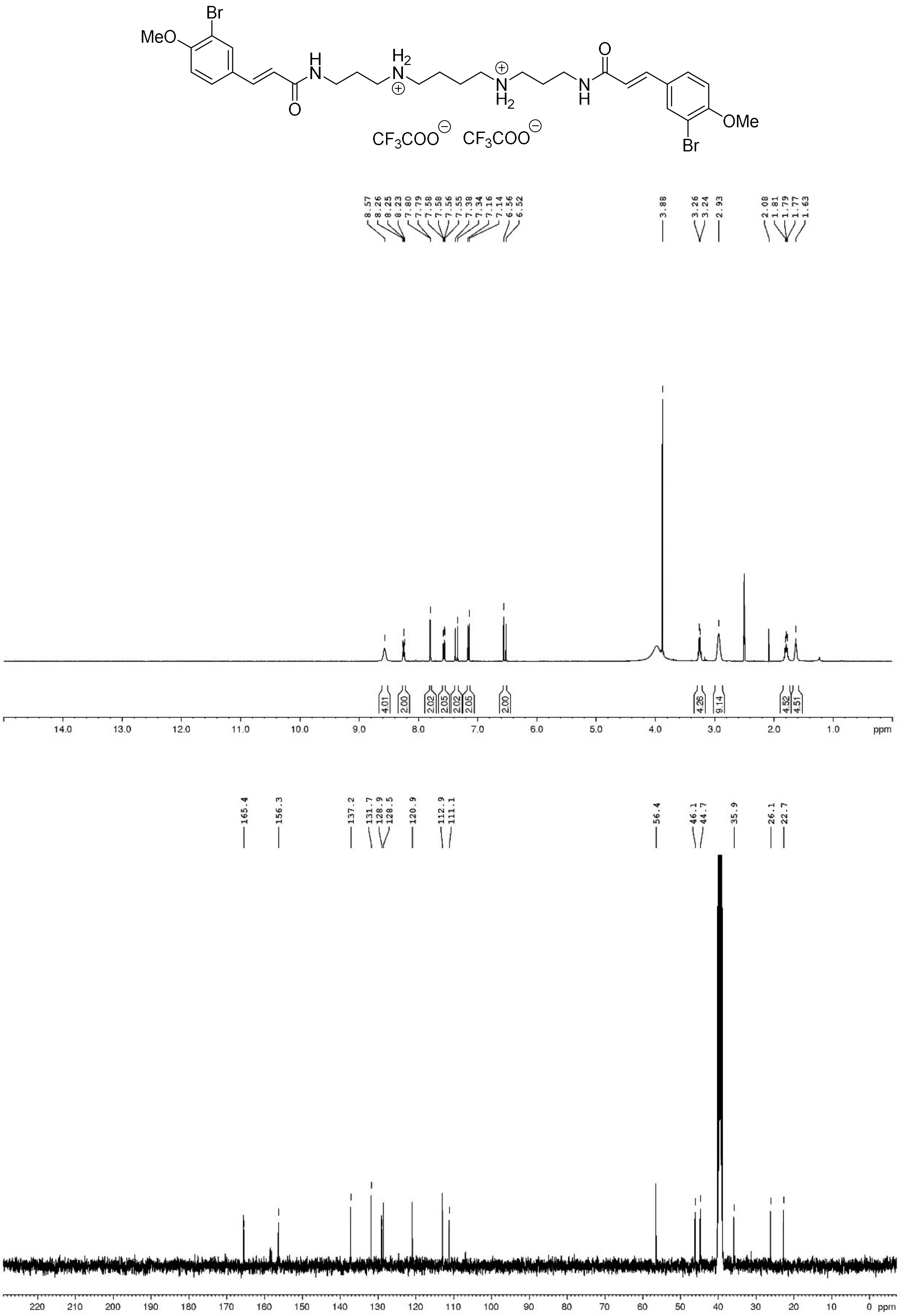

Figure S13. ^{1}H (DMSO-d_6, 400 MHz) and ^{13}C (DMSO-d_6, 100 MHz) NMR spectra for **15a**.

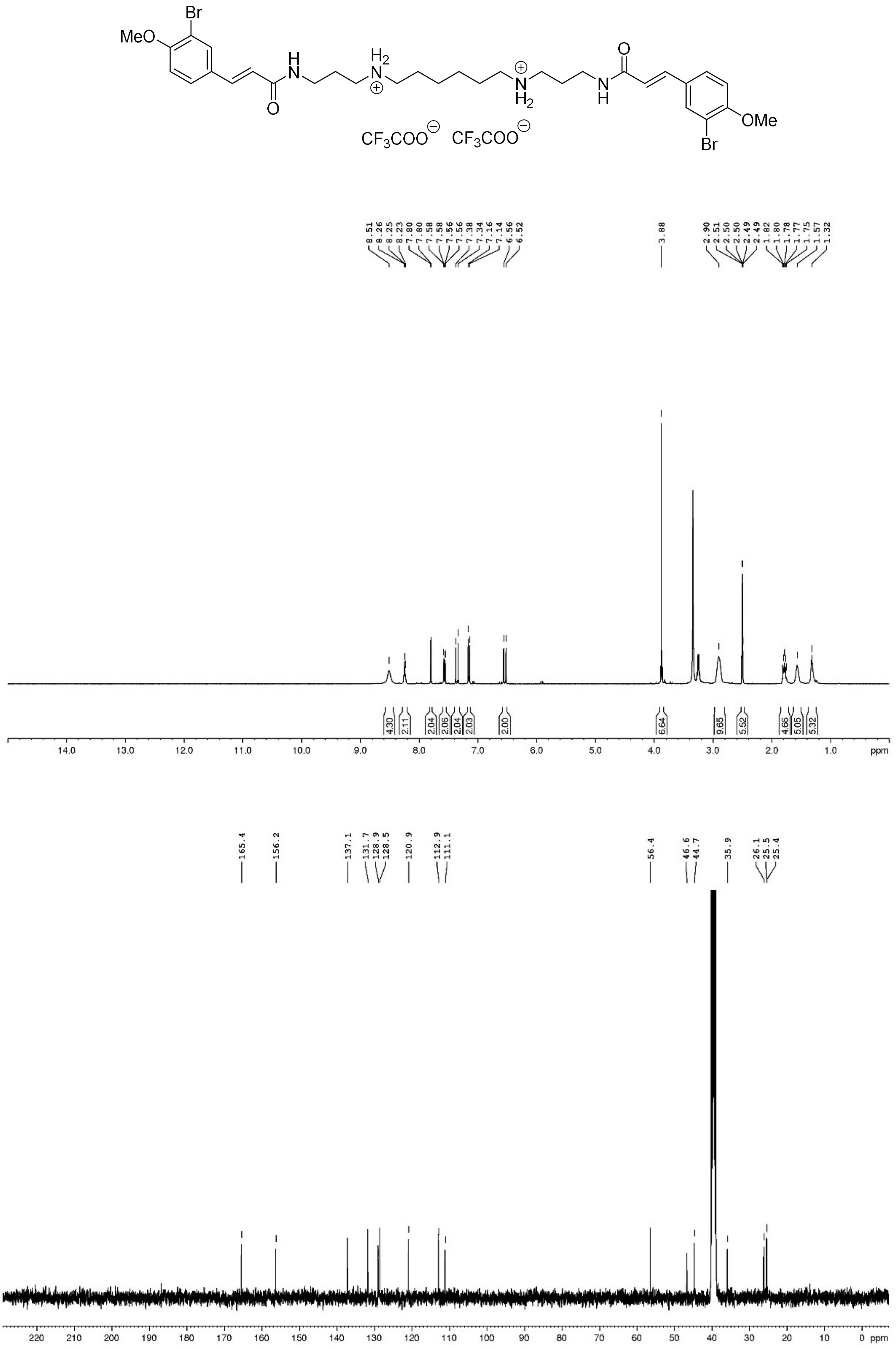

Figure S14. ^{1}H (DMSO-d_6, 400 MHz) and ^{13}C (DMSO-d_6, 100 MHz) NMR spectra for **15b**.

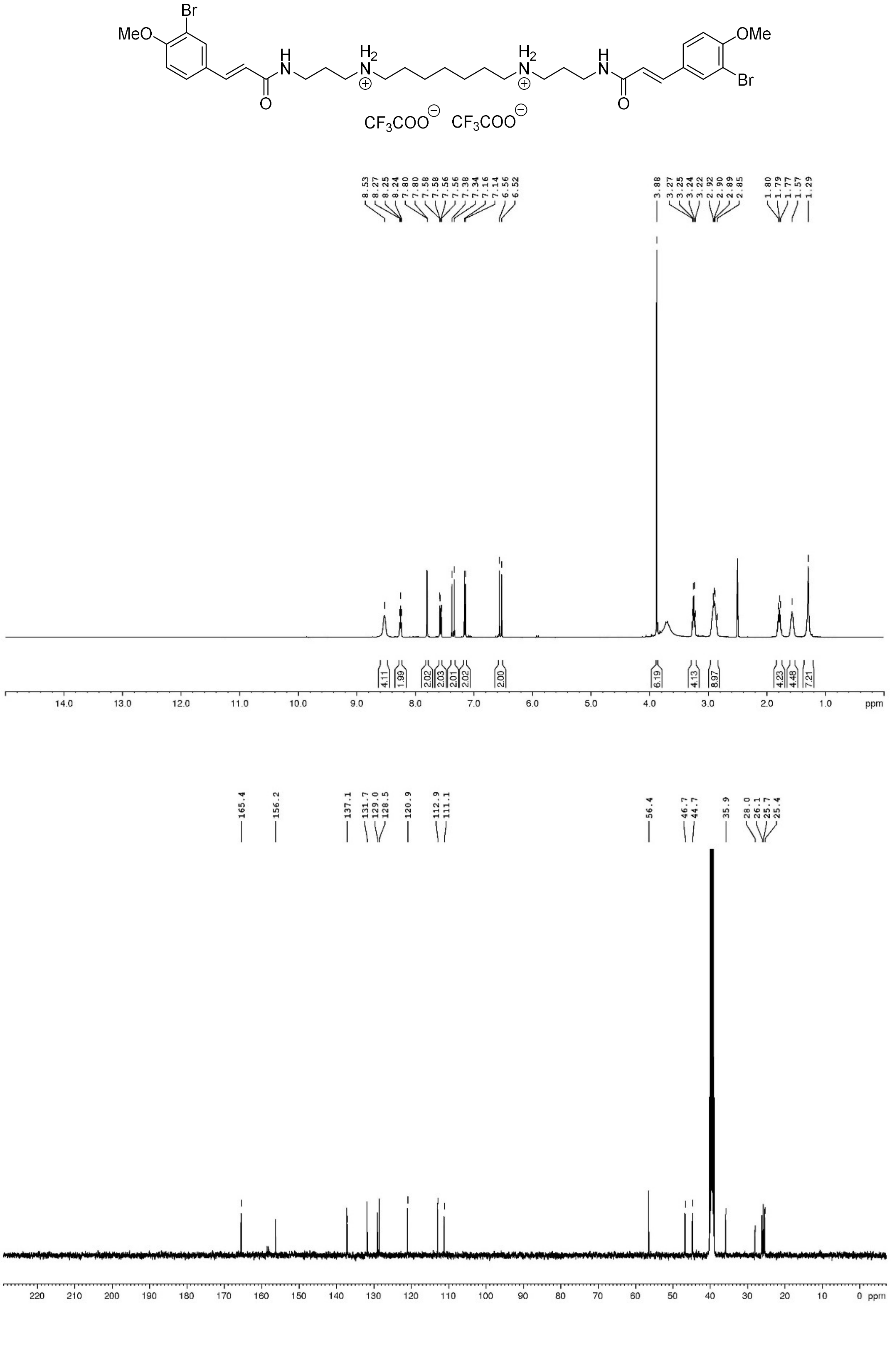

Figure S15. ^{1}H (DMSO-d_6, 400 MHz) and ^{13}C (DMSO-d_6, 100 MHz) NMR spectra for **15c**.

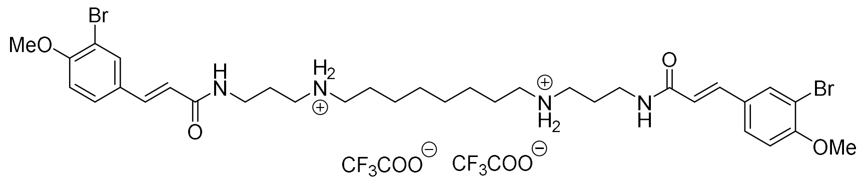

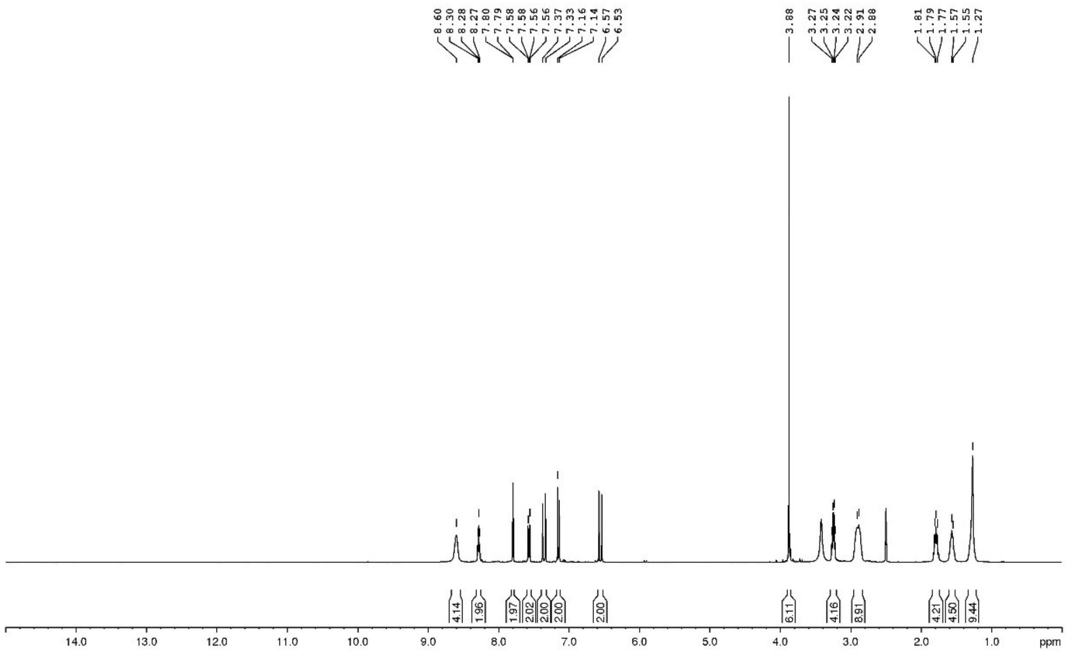

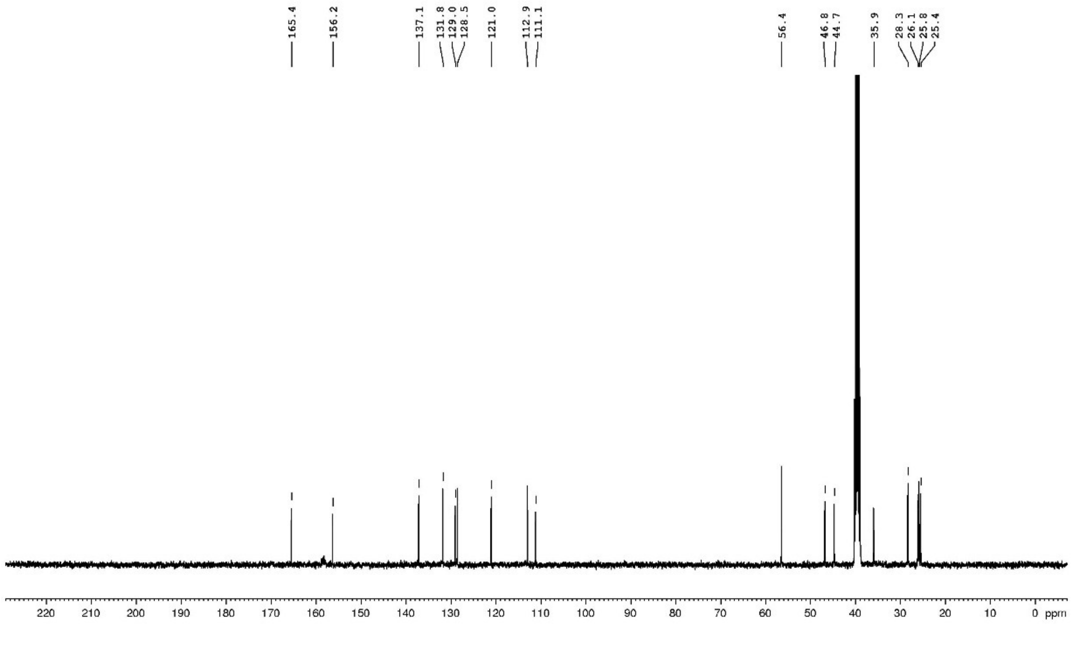

Figure S16. ^{1}H (DMSO-d_6, 400 MHz) and ^{13}C (DMSO-d_6, 100 MHz) NMR spectra for **15d**.

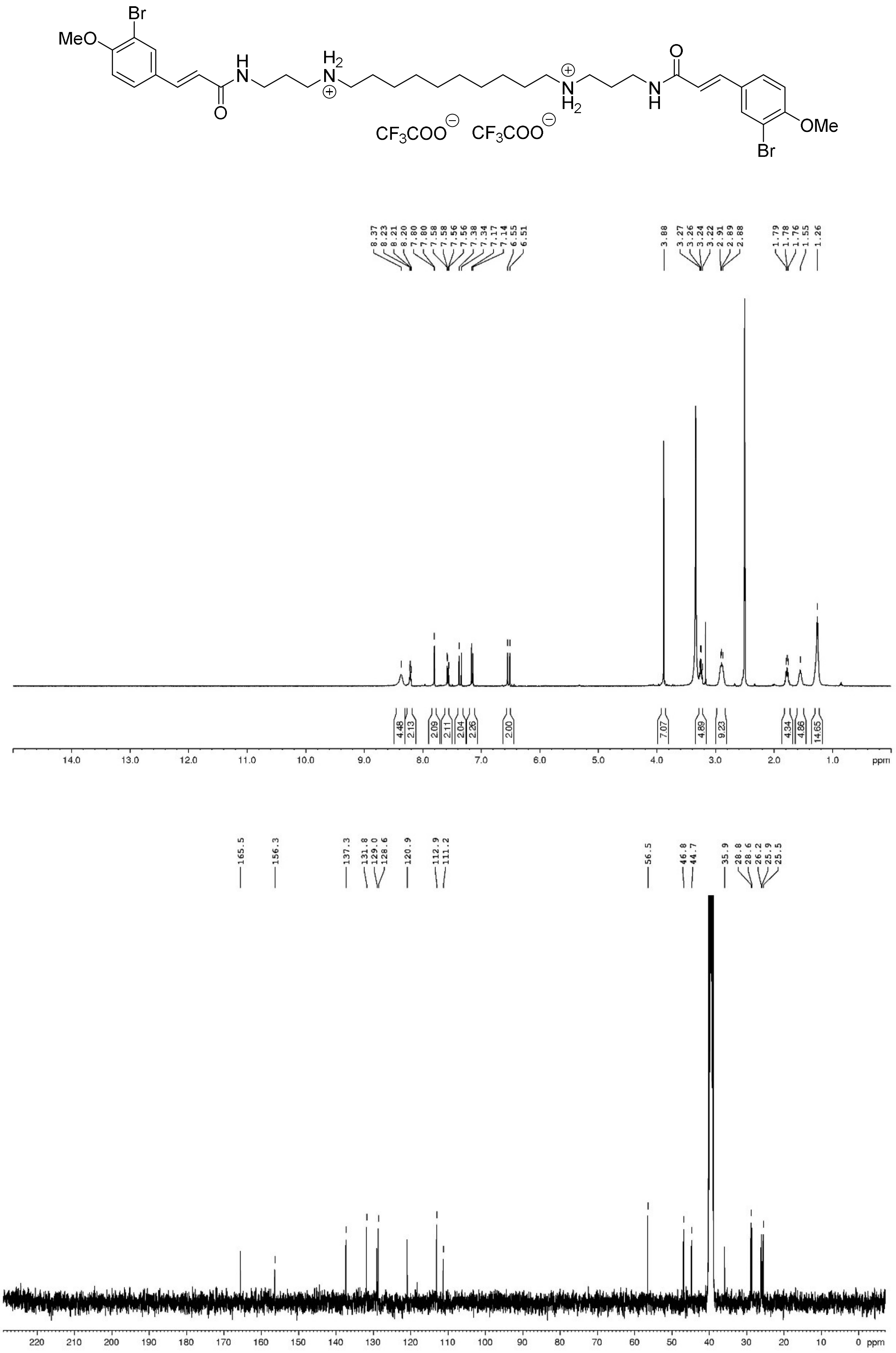

Figure S17. ^{1}H (DMSO-d_6, 400 MHz) and ^{13}C (DMSO-d_6, 100 MHz) NMR spectra for **15e**.

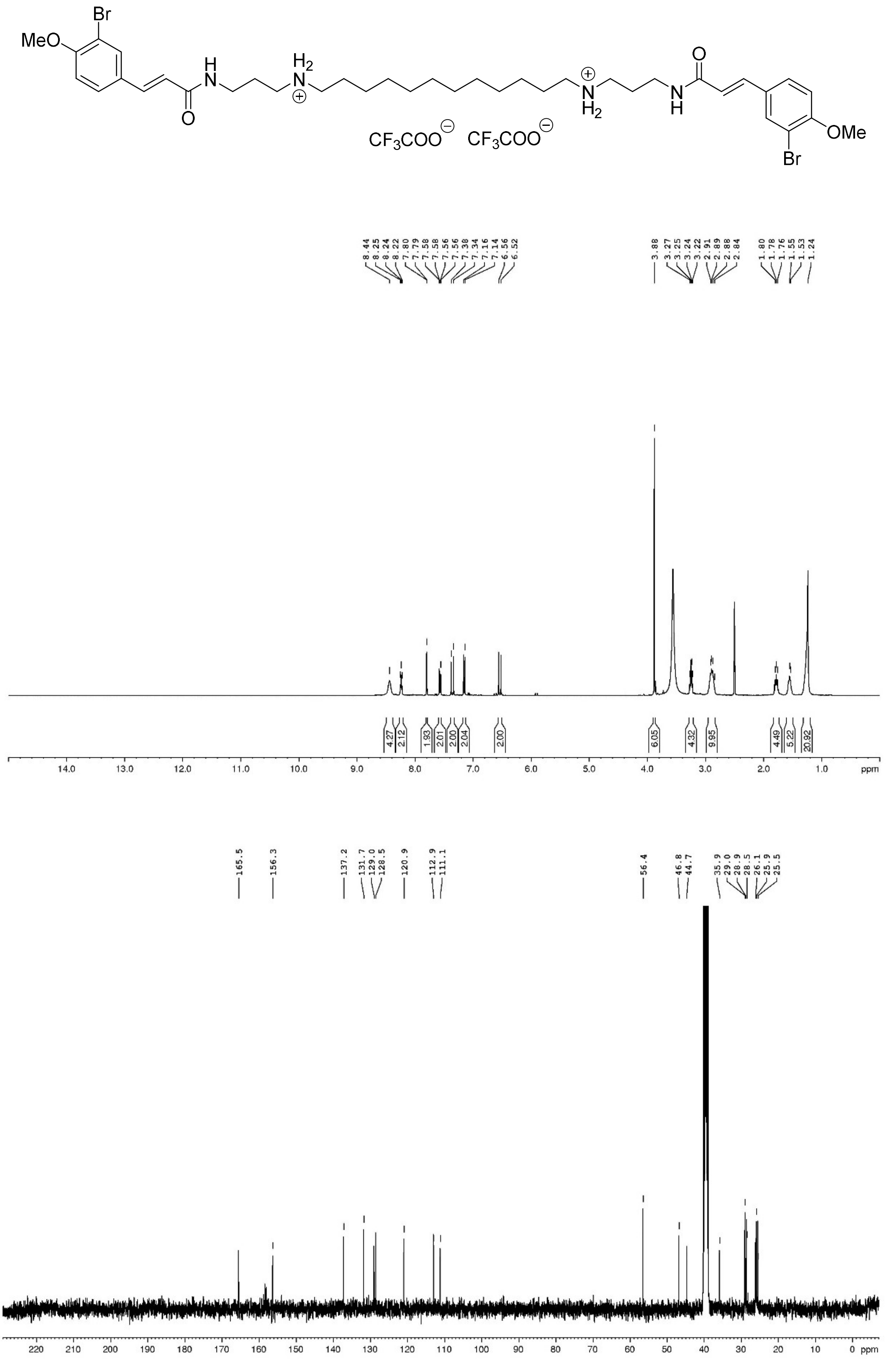

Figure S18. ^{1}H (DMSO-d_6, 400 MHz) and ^{13}C (DMSO-d_6, 100 MHz) NMR spectra for **15f**.

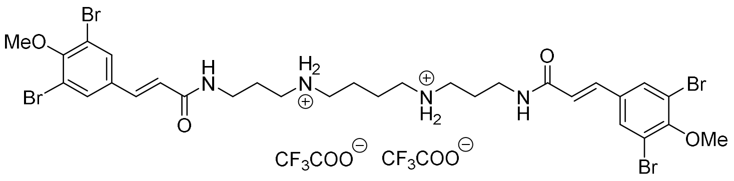

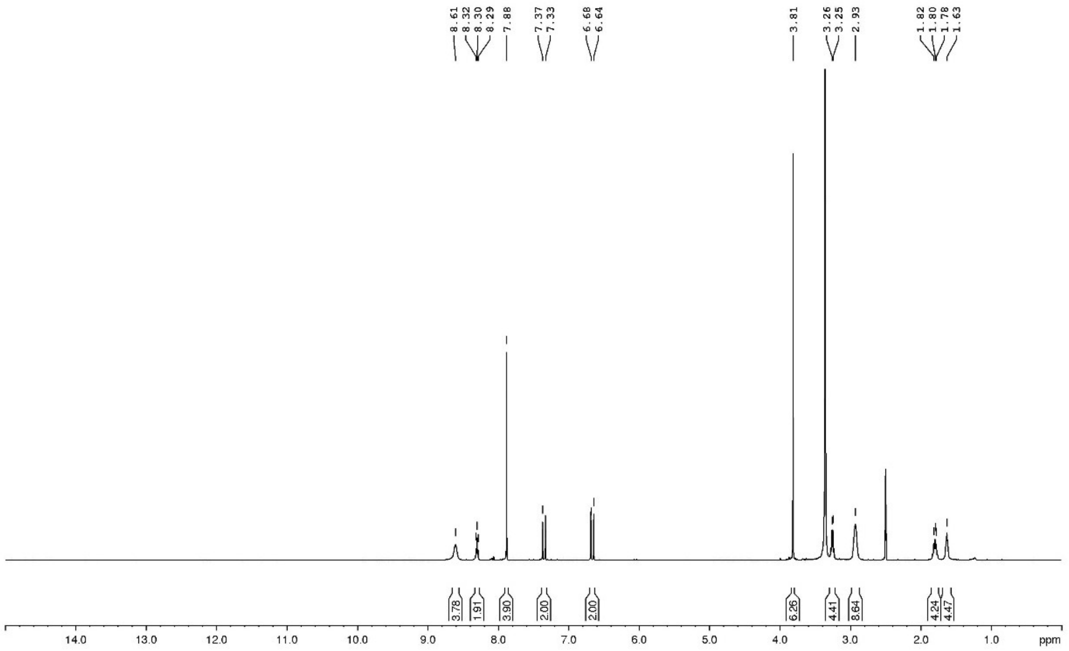

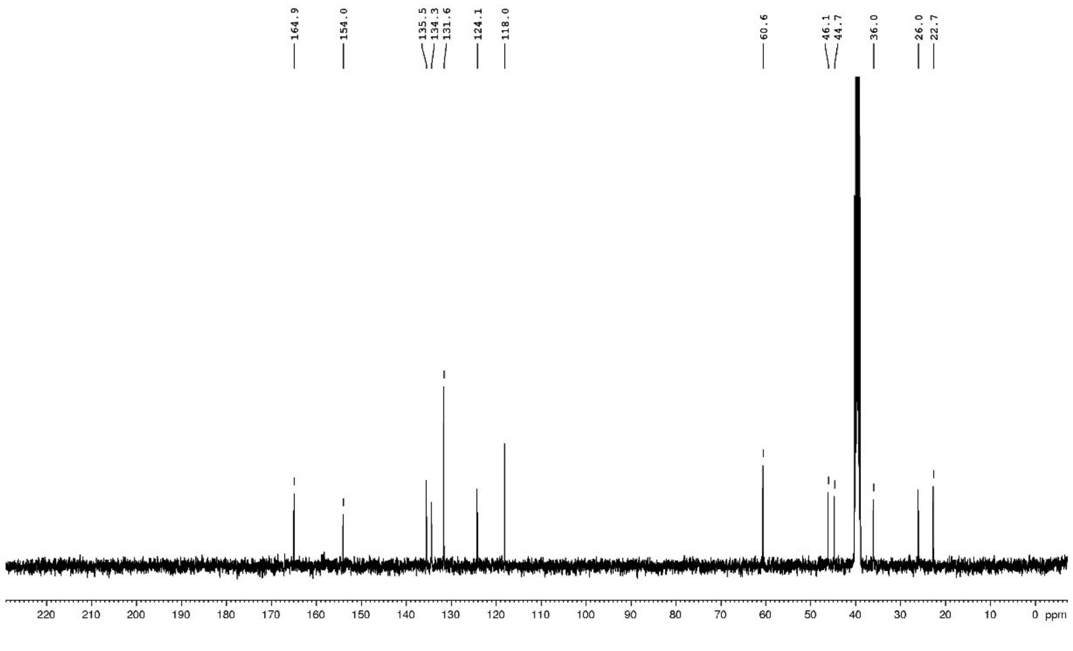

Figure S19. ^{1}H (DMSO-d_6, 400 MHz) and ^{13}C (DMSO-d_6, 100 MHz) NMR spectra for **16a**.

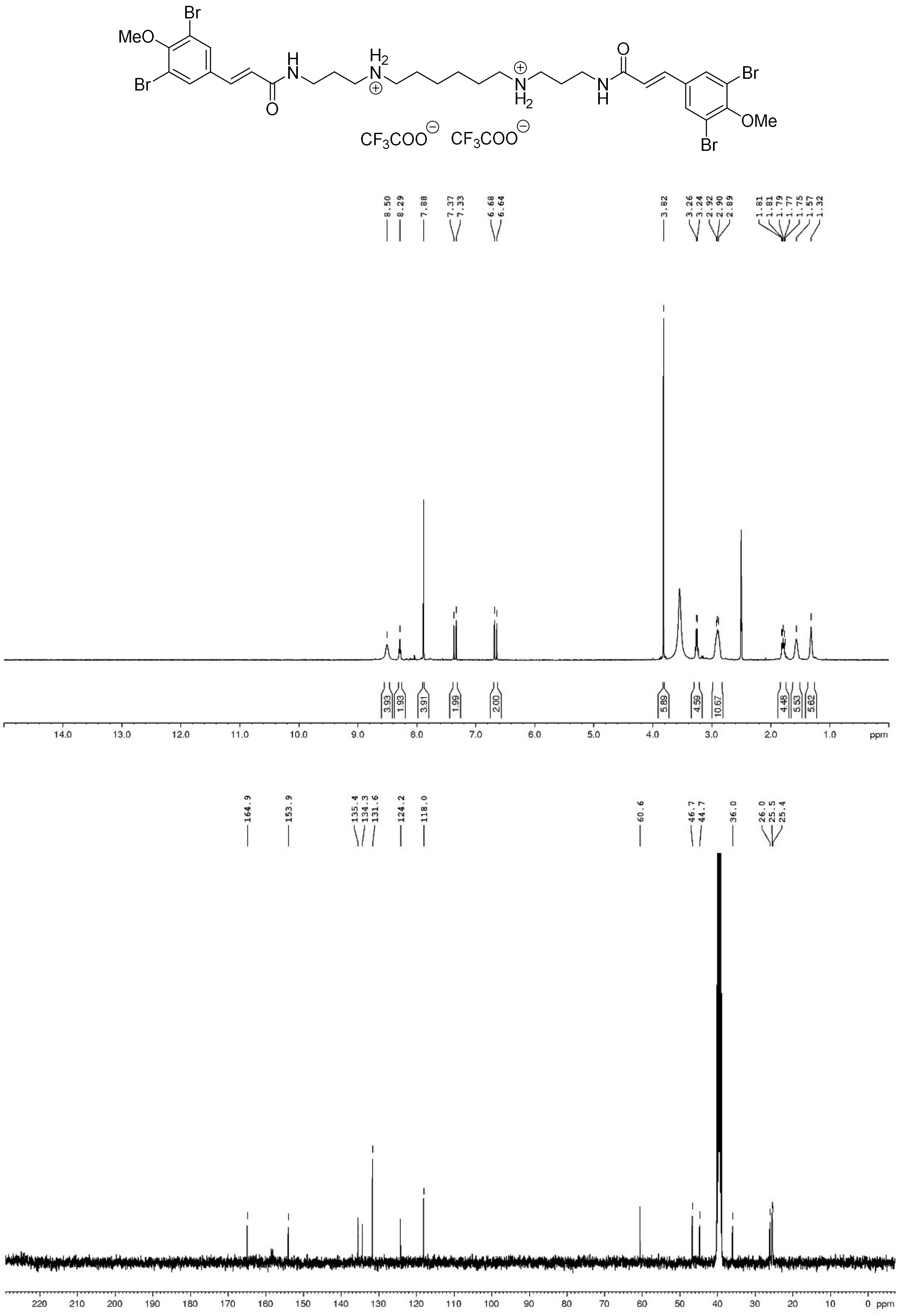

Figure S20. ^{1}H (DMSO-d_6, 400 MHz) and ^{13}C (DMSO-d_6, 100 MHz) NMR spectra for **16b**.

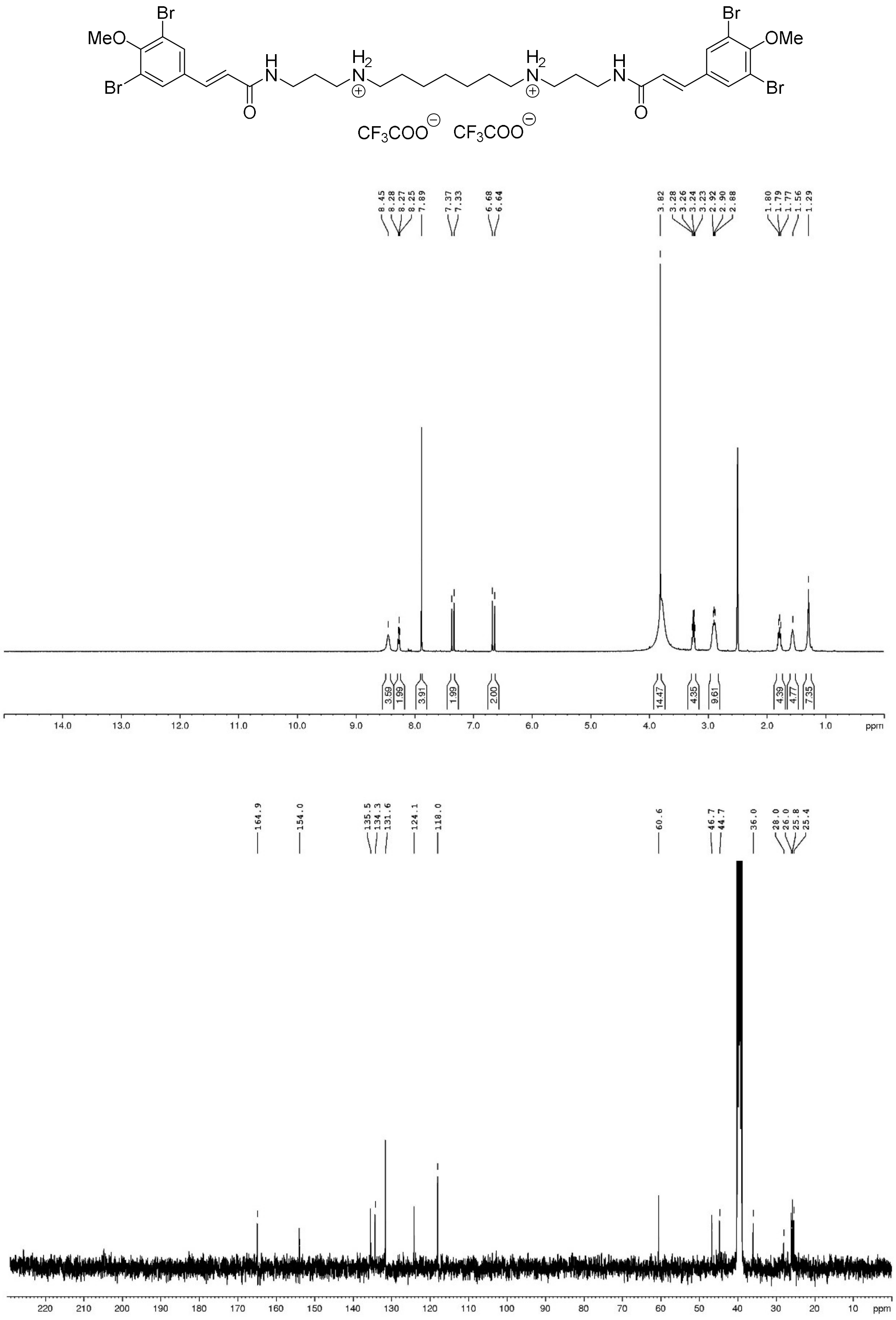

Figure S21. ^{1}H (DMSO-d_6, 400 MHz) and ^{13}C (DMSO-d_6, 100 MHz) NMR spectra for **16c**.

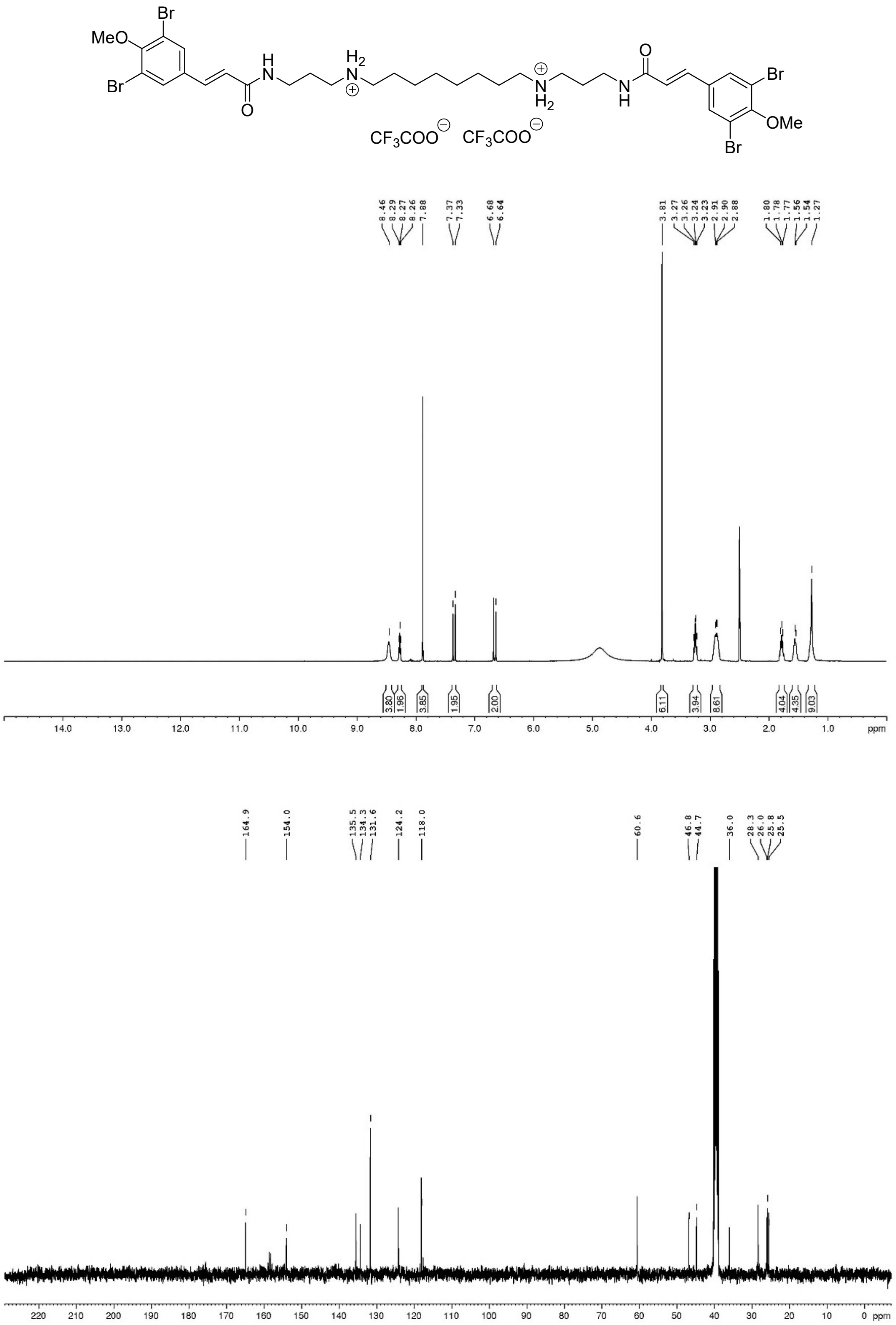

Figure S22. ^{1}H (DMSO-d_6, 400 MHz) and ^{13}C (DMSO-d_6, 100 MHz) NMR spectra for **16d**.

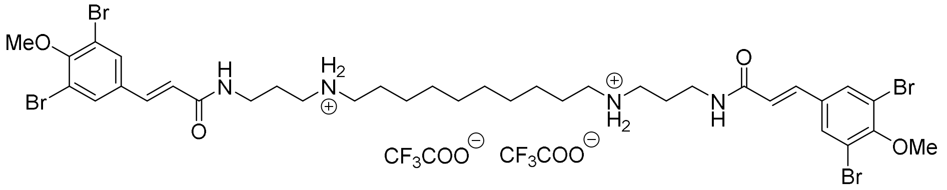

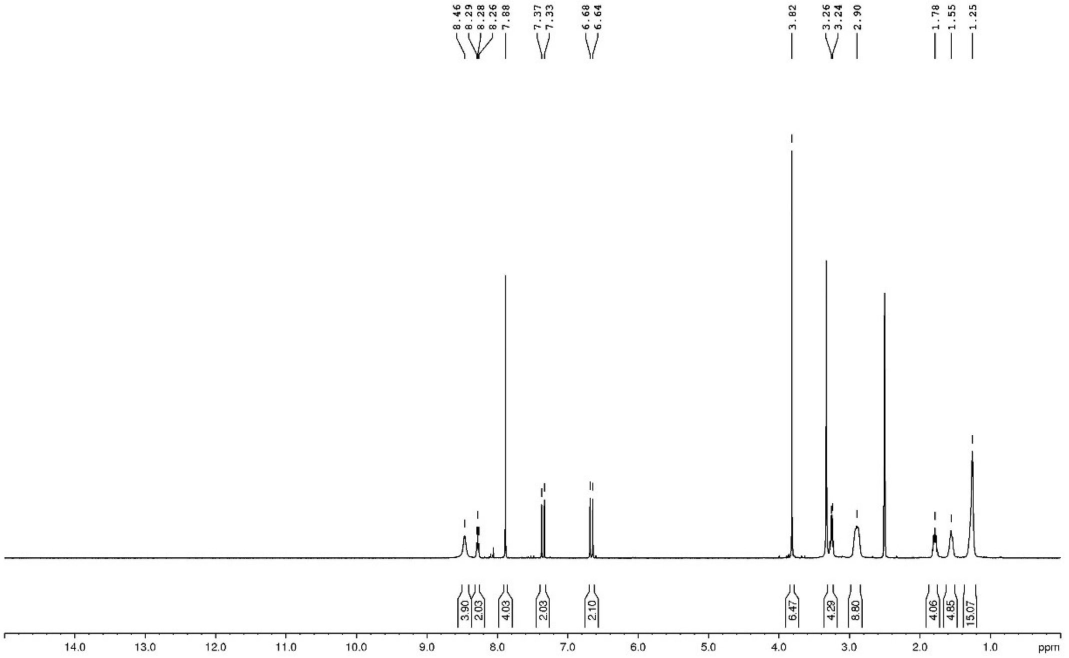

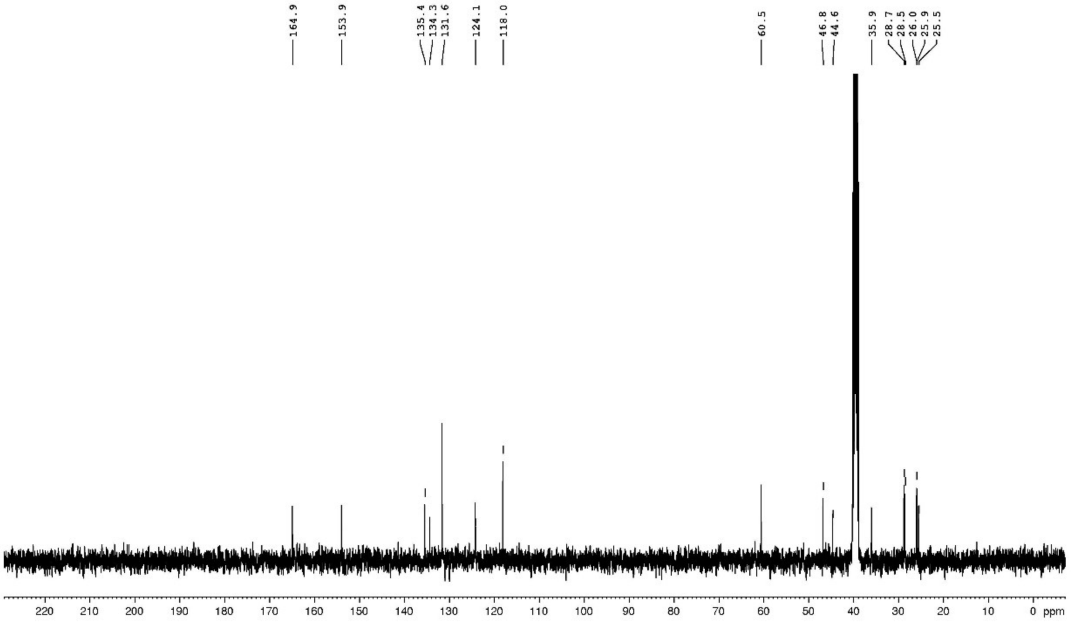

Figure S23. ^{1}H (DMSO-d_6, 400 MHz) and ^{13}C (DMSO-d_6, 100 MHz) NMR spectra for **16e**.

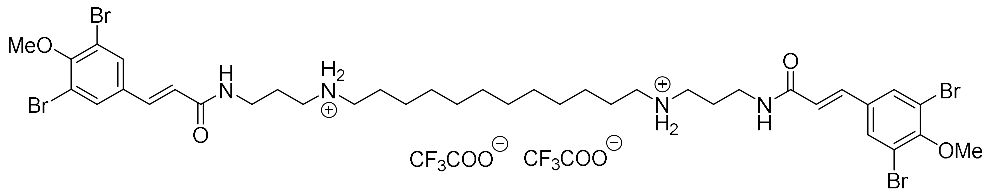

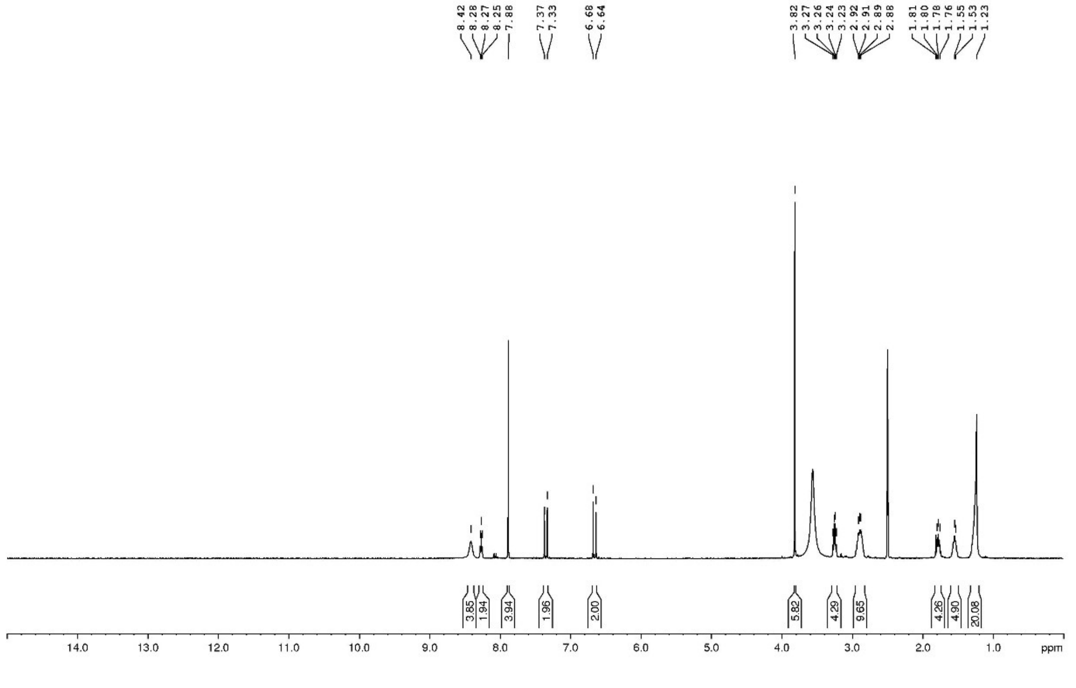

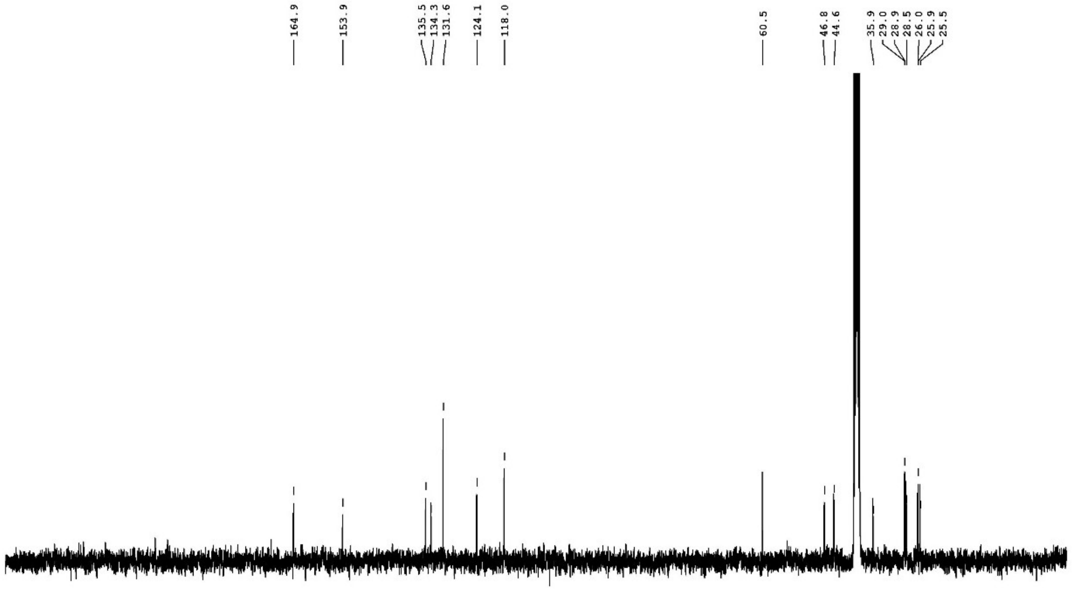

Figure S24. ^{1}H (DMSO-d_6, 400 MHz) and ^{13}C (DMSO-d_6, 100 MHz) NMR spectra for **16f**.
